# Computational Design of Miniproteins as SARS-CoV-2 Therapeutic Inhibitors

**DOI:** 10.3390/ijms23020838

**Published:** 2022-01-13

**Authors:** Bahaa Jawad, Puja Adhikari, Kun Cheng, Rudolf Podgornik, Wai-Yim Ching

**Affiliations:** 1 Department of Physics and Astronomy, University of Missouri-Kansas City, Kansas City, MO 64110, USA; bajrmd@mail.umkc.edu (B.J.); paz67@umkc.edu (P.A.); 2 Department of Applied Sciences, University of Technology, Baghdad 10066, Iraq; 3 Division of Pharmacology and Pharmaceutical Sciences, School of Pharmacy, University of Missouri-Kansas City, Kansas City, MO 64108, USA; chengkun@umkc.edu; 4 Wenzhou Institute of the University of Chinese Academy of Sciences, Wenzhou 325000, China; rudolf.podgornik@fmf.uni-lj.si; 5 School of Physical Sciences and Kavli Institute of Theoretical Science, University of Chinese Academy of Sciences, Beijing 100049, China; 6 CAS Key Laboratory of Soft Matter Physics, Institute of Physics, Chinese Academy of Sciences, Beijing 100090, China

**Keywords:** SARS-CoV-2, therapeutics design, miniprotein inhibitors modification, molecular dynamics simulation, density functional calculation

## Abstract

A rational therapeutic strategy is urgently needed for combating SARS-CoV-2 infection. Viral infection initiates when the SARS-CoV-2 receptor-binding domain (RBD) binds to the ACE2 receptor, and thus, inhibiting RBD is a promising therapeutic for blocking viral entry. In this study, the structure of lead antiviral candidate binder (LCB1), which has three alpha-helices (H1, H2, and H3), is used as a template to design and simulate several miniprotein RBD inhibitors. LCB1 undergoes two modifications: structural modification by truncation of the H3 to reduce its size, followed by single and double amino acid substitutions to enhance its binding with RBD. We use molecular dynamics (MD) simulations supported by ab initio density functional theory (DFT) calculations. Complete binding profiles of all miniproteins with RBD have been determined. The MD investigations reveal that the H3 truncation results in a small inhibitor with a −1.5 kcal/mol tighter binding to RBD than original LCB1, while the best miniprotein with higher binding affinity involves D17R or E11V + D17R mutation. DFT calculations provide atomic-scale details on the role of hydrogen bonding and partial charge distribution in stabilizing the minibinder:RBD complex. This study provides insights into general principles for designing potential therapeutics for SARS-CoV-2.

## 1. Introduction

Spike (S) protein of severe acute respiratory syndrome (SARS-CoV-2) virus is an ideal molecular target to develop prophylactics and therapeutics against the ongoing COVID-19 pandemic [1,2,3,4,5]. In particular, the receptor-binding domain (RBD) of S-protein initiates the interaction with a human cell angiotensin-converting enzyme 2 (ACE2) receptor during the infection process [6,7]. Thus, targeting the RBD is a promising therapeutic approach for preventing viral uptake.

Several antiviral strategies have been proposed to block S-protein SARS-CoV-2 entry such as new drugs or repurposing the existing ones [4,8], natural or engineered neutralizing antibodies [9,10,11,12], small peptides [13,14], but are all hindered by many challenges. For instance, the drug discovery process is inherently slow to fulfill the urgent needs for fast therapeutic solutions [15,16]. Other traditional options, such as antibodies, while being effective treatments, also imply several concerns. First, the therapeutic antibodies are associated with potential risks or limitations such as antibody-dependent enhancement (ADE) of COVID-19 [17,18]. Second, the antibody’s efficacy may be reduced due to the development of antibody resistance caused by the rapid accumulation of S-protein escape mutations [19]. Finally, they are not suitable for intranasal delivery since they are large and often not extremely stable molecules in addition to having a lower binding affinity [20]. On the other hand, vaccinations are the most successful therapeutics [21,22], but alternative treatments are necessary in some cases such as when certain patients are unable to get a vaccination owing to their medical condition, lack of availability, or when vaccine efficacy is compromised by new SARS-CoV-2 variants [23]. Besides these concerns, the vaccine candidates and the antibodies rely on the molecular mechanism to interact with pathogens in a way that radically differs from how the pathogen binds its host targets [24].

The design of de novo proteins to neutralize RBD of SARS-CoV-2 is a new therapy that has key advantages over natural antibodies [20,24]. These de novo miniprotein inhibitors or ACE2 decoys have no associated ADE risks and are intrinsically resilient to viral mutation escape. They have a high binding affinity and selectivity to RBD S-protein. They also possess high thermostability and solubility that enables direct delivery to the nasal or respiratory system. In addition, they are also easy to store without refrigeration. Cao et al. have used two de novo computational approaches to design synthetic miniprotein (MP) inhibitors and succeeded in designing two leading candidates named LCB1 and LCB3 with high binding affinity to the SARS-CoV-2 RBD and high neutralizing ability [20]. However, these MPs are still considered as large-size inhibitors [25], with LCB1 and LCB3 having 56 and 64 residues, respectively. In this regard, designing a small-size MP would be more desirable since it will lower manufacturing costs with higher output and easier penetration into tissues and cells with high specificity. On the other hand, the dissociation constants (K_D_) for the binding of LCB1 and LCB3 to RBD using the experimental biolayer interferometry (BLI) technique could not accurately be determined because of a lack of instrument sensitivity and long equilibration times below 200 pM [20]. Therefore, further investigations are necessary to understand their binding modes and general principles guiding the new antiviral design. To gain further insights into such binding processes at the molecular, amino acid (AA), and atomic levels, computational approaches specifically involving MD simulations and ab initio quantum chemical calculations have been implemented.

The present study aims to systematically design and develop a more effective MP, with small size and high binding affinity, to inhibit RBD SARS-CoV2. To achieve this goal, LCB1 is used as a template and subjected to two stages of modification: structural alteration and AA substitutions at specific positions. In the first stage, the alpha-helix 3 (H3) of LCB1 is truncated without changing its secondary structure. This follows by a point and a double AA substitution at a certain position to increase its binding to RBD. Our study addresses detailed thermodynamics and binding mechanism for SARS-CoV-2 RBD binding MPs such as LCB1, LCB3, and the derived MPs from the original LCB1. We attempt to obtain the complete interaction free energy profile of the binding mechanisms including the total binding free energy (BFE) and its thermodynamic components. The BFE is decomposed in terms of amino acid residues to ascertain the effect of mutations. Furthermore, detailed interatomic bonding including hydrogen bonding (HB) and the AA–AA interactions as well as the partial charge distributions are addressed using ab initio DFT calculations.

In order to reach these goals, we adopt two well-tested methodologies: classical MD simulations and ab initio quantum chemical calculations. MD simulations are performed for fifteen different models of the minibinder:RBD complex on μs timescale. The AMBER (Assisted Model Building with Energy Refinement) package within the context of an explicit TIP3P water solvent model is used [26]. This is followed by molecular mechanics generalized Born surface area (MM-GBSA) method for BFE calculations. The supporting DFT calculations are carried out on smaller size models for further structural elucidation based on the structures optimized using the Vienna ab initio simulation package (VASP) [27,28] and followed by the orthogonalized linear combination of atomic orbitals (OLCAO) method [29].

## 2. Results

### 2.1. Binding of LCB1 and LCB3 to RBD SARS-CoV-2

Since the BLI experiment assay cannot predict K_D_ below 1 nM for LCB3:RBD and LCB1:RBD complexes [20], this motivated us to calculate their BFEs using the MM-GBSA method at a neutral pH value and 0.15 M univalent NaCl salt concentration. The MD simulations in the explicit TIP3P water model have been performed across 1 μs timescale at T = 310 K (37 °C) for all models (See Extended method section in Supplementary Materials). The root mean square deviation (RMSD) and root mean square fluctuation (RMSF) of M1-MD and M2-MD simulations are presented and discussed in Supplementary Materials (Figures S1 and S2). Our results indicate a large conformational change in RBD at its loop 3 (T470 to P491) in model M1-MD, as can be seen in the RMSD and RMSF values after 60 ns in Figures S1a and S2a, respectively. Figure S3 shows the convergence plot of the BFE as a function of 10 successive MD. The length of each MD run is 100 ns, 1000 ns in total (10 × 100 ns) (see Supplementary Materials). BFEs in all models achieve convergence, as evidenced by a slow fluctuation around the mean value of the BFE with standard deviation (SD) less than 10.5 and 8.1 kcal/mol for M1-MD and M2-MD, respectively.

Table 1 lists the net BFE (ΔG_bind_) for M1-MD and M2-MD with their thermodynamic components. Both LCB1 and LCB3 bind strongly to RBD with ΔG_bind_ of −28.73 and −25.48 kcal/mol, respectively. These values are tighter than the BFE of −12.86 kcal/mol reported in [30], indicating that the binding of MPs to RBD has sufficient affinity to compete with the binding of RBD to ACE2. Interestingly, the binding of LCB1 with RBD is more favorable than LCB3 by −3.25 kcal/mol, mainly from the favorable ΔG_ele_. The decomposed binding properties behave similarly in M1-MD and M2-MD in which ΔE_vdW_, ΔE_ele_, and ΔG_SA_ contribute favorably to BFE to counteract the unfavorable contributions from dehydration (ΔG_GB_) and entropy (−TΔS). We conclude that the long-range Coulombic electrostatic interaction (ΔE_ele_) plays a pivotal role in the MPs recognition process with RBD while the attractive component of the van der Waals interaction (ΔE_vdW_) is the main factor to stabilize the complex. This is consistent with what is observed in the RBD:ACE2 complex [30].

**Figure 1:**
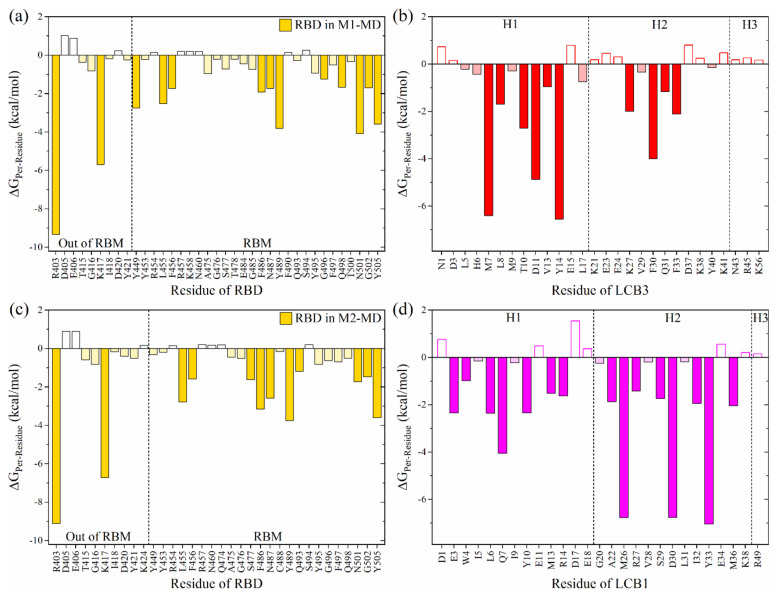
Per-residue interaction spectrum of miniprotein:RBD complexes: (**a**,**b**) for M1-MD. (**c**,**d**) for M2-MD. The left panels are for RBD residues while the right panels are for miniprotein residues. The filled chart bars are for significant AAs (ΔG_Per-residue_ ≤ −1 kcal/mol), while those with light color represent the favored AAs (ΔG_Per-residue_ ≥ −1 to ≤ −0.15 kcal/mol). The unfilled bars are for unfavorably interacting AAs (ΔG_Per-residue_ ≥ 0.15 kcal/mol).

To identify the key interacting residues, the per-residue BFE decompositions have been performed and shown in Figure 1a,c for interaction spectrums of RBD in M1-MD and M2-MD, respectively. The observation can be summarized as follows: (i) The key interacting residues in RBD differ slightly, indicating that their binding modes are relatively distinct. (ii) There are 37 (34) interacting residues in RBD in M1-MD (M2-MD) but with only 9 (10) located outside the RBM. (iii) There are 10 common AAs in both models (R^403^, K^417^, L^455^, F^456^, F^486^, N^487^, Y^489^, N^501^, G^502^ and Y^505^) with significant interaction. The first two AAs (R^403^ and K^417^) have the most attractive ΔG_Per-Residue_ of −9.2 and −6 kcal/mol, respectively. The contributions from these residues to RBD:ACE2 complex are only 0.25 and −1.46 kcal/mol [30]. Therefore, the highest binding affinity of LCB3:RBD or LCB1:RBD vs. SARS-CoV-2 RBD:ACE2 complex can be traced to these two AAs. K^417^ has been identified to play a critical role in enhancing the binding of RBD SARS-CoV-2 with ACE2 as compared to SARS-CoV [30,31,32]. The other eight AAs have also been reported as key interacting AAs in RBD:ACE2 complex [30,31,33,34]. These results demonstrate that the MPs at this binding site can obstruct the binding between RBD and ACE2.

On the other hand, the interaction spectrums of LCB3 (MP1) and LCB1 (MP2) are shown in Figure 1b,d. The important interacting residues on LCB3 with ΔG_Per-residue_ ≤ −1 kcal/mol are M^7^, L^8^, T^10^, D^11^, V^13^, Y^14^, K^27^, F^30^, Q^31^, and F^33^, while those on LCB1 are E^3^, W^4^, L^6^, Q^7^, Y^10^, M^13^, R^14^, A^22^, M^26^, R^27^, S^29^, D^30^, I^32^, Y^33^, and M^36^. The contributions of LCB3 and LCB1 to total BFE in the complex formation are 40% and 50%, respectively, revealing that the LCB1 binds more selectively to RBD. Another main critical finding is there is no significant contribution from the H3 in both complexes. This raises a crucial question: Is it possible to design a small miniprotein with a better binding profile than the original ones? To address this question, we design a new miniprotein derived from LCB1 by truncating its H3. This is discussed in the following section.

### 2.2. Structural Modification on LCB1

Smaller size proteins have several advantages as in reduced production costs with high output, low side effects, high selectivity, etc. [35,36]. Cao et al. identified LCB1 as a smaller miniprotein with 56 AAs [20], but it is still considered to be a large inhibitor [25]. Therefore, this subsection emphasizes the role of structural modification on LCB1 to design a smaller miniprotein with a better binding profile. We derive MP3 from LCB1 (MP2) by truncating its H3 (see Section 4). Note that the binding mode of MP3 with RBD is the same as that of the LCB1:RBD complex. To validate this hypothesis, a docking approach of the MP3 with RBD is employed using the ZDOCK web server [37]. Residues 401 to 508 on RBD are selected as a binding site of RBD while all other residues of MP3 are chosen as contacting residues. The best-predicted structures of the MP3:RBD from ZDOCK closely match those in the LCB1:RBD complex (Figure S4). This confirms that the designed model MP3:RBD from the original LCB1:RBD is correct.

To verify the structural stability of binding MP3 with RBD, their RMSDs are monitored during MD simulation as shown in Figure S1c. They are stable and have the same range as in M2-MD (LCB1:RBD), demonstrating that H3 truncation of LCB1 has no impact on its structural features. This is also confirmed by the lower values of RMSFs (Figure S2e,f). Moreover, we calculate the secondary structure of MP3 across the entire simulation by using the Define Secondary Structure of Proteins (DSSP) algorithm implemented in CPPTRAJ program of AMBER [38,39], showing that the α-helices of MP3 are preserved over the simulation (Figure S5).

We now turn to BFE analysis. Table S1 shows that the predicted ΔG_bind_ of M3-MD is −30.2 kcal/mol, stronger than the BFE of M2-MD by −1.46 kcal/mol. This preferred binding can be mainly attributed to ΔG_ele_ (Table S1 vs. Table 1). The solubility of MP3 and LCB1 are found to be similar (Figure S6) [40]. Overall, the binding and solubility of MP3, together with its size, suggest that it could be a promising RBD inhibitor.

To further check the thermostability of MP3, we used the SCooP webserver for predicting thermodynamic quantities that characterize the folding process including the change in enthalpy (ΔH_m_) and in heat capacity (ΔC_p_) upon folding, the melting temperature (T_m_) and the folding free energy (ΔG_r_) at room temperature [41]. In this analysis, 10 structures including only MPs from the last step of each of the 10 subsequent MDs have been extracted. Figure S7 shows these quantities. First, the predicted T_m_ values for MP1 and MP2 are 79 ± 4 and 77 ± 2 °C respectively, which are in relatively reasonable agreement with the experimental values of greater than 95 °C [20]. Surprisingly, the T_m_ of MP3 is 90 ± 2.5 °C higher than that of MP2, indicating the truncation of H3 from MP2 increasing its thermostability. Additionally, the ΔH_m_ and ΔC_p_ of MP3 are both greater than those of MP2, whereas the opposite trend exists in ΔG_r_. Here, further investigation is necessary.

Even though the MP3 does not contain H3, it has the same interaction spectrum and the total contribution to BFE (50%) is the same as in LCB1 (Figure S8a,b). Some interacting AAs in MP3 contribute unfavorably to BFE such as D^1^, K^2^, E^11^, D^17^, E^18^, E^34^, K^38^, and D^40^ (unfilled bars in Figure S8b). These AAs carry charges and require high desolvation energy, especially ΔG_GB_, leading to unfavored interactions for complex formation. We propose that exchanging these AAs by other carefully selected AAs may boost the binding affinity of MP3 with RBD. It should be mentioned that D^1^, K^2^, K^38^, and D^40^ are located on the end termini of MP3 and E^18^ is at the turn between H1 and H2, so we kept them unchanged. Since the solubility is affected by the number of charged or polar AAs, changing these charged AAs to hydrophobic AAs reduces solubility. To maintain the solubility of the derivative inhibitor, we perform AA substitutions at only two positions E^11^ and D^17^ (see Section 4) to be described in the next subsection.

### 2.3. Amino Acid Substitutions on MP3

To enhance the binding profile of MP3 while maintaining its solubility, we make single or double substitutions at positions 11 and 17 (see Section 4). Briefly, twelve models are designed: Ten for a single mutation. The single mutations that enhanced binding are then mixed to generate models for double mutation. Model M3-MD is used as a control to compare the BFE of its derivative inhibitors.

**Figure 2:**
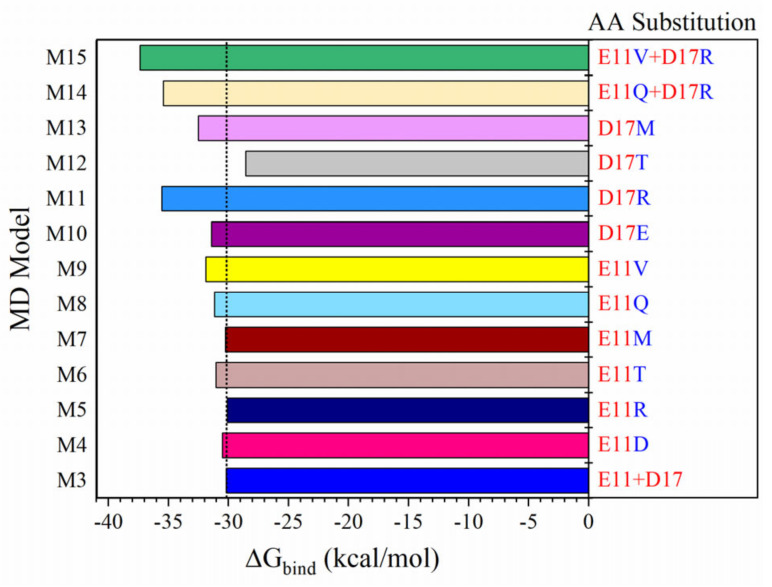
The impact of single and double AA substituting on the MP3 binding to RBD SARS-CoV-2. M3 used as a control. The residue positions that are replaced (in red letters) to other AAs (blue letters).

Figure 2 shows the effect of substituting the single or double residues at positions 11 and 17 on MP3 binding. Single mutations at position 11 do not increase the binding affinity when changing from negatively charged AA (E^11^) to other charged AAs (D or R) or to a relatively large hydrophobic AA (M) but replacing position 11 with polar AAs (T or Q) or small hydrophobic AA (V is smaller than M) results in enhanced binding affinity of MP3. In general, ΔG_ele_, particularly ΔG_GB_, plays a role in this optimization (Table S1). V and Q are the most preferred AAs at position 11 with ΔΔG of −1.69 and −1.0 kcal/mol, respectively, compared to M3-MD. The mutations at position 17 are crucial because this AA can make non-covalent contact with AAs in RBD at the interface. The D17T mutation tends to reduce the binding with RBD, but the other three mutations (D17E, D17R, and D17M) have the opposite trends because their sidechains are longer than D, thus promoting more interactions with the RBD. This is supported by the more favored ΔE_vdW_ in their corresponding models (M10, M11, and M13) relative to M3-MD (Table S1). The D17R mutation in M11-MD has a stronger binding of −5.36 kcal/mol than M3-MD (Table S1 or Figure 2). So, in attempting the double mutation, R^17^ is retained. The predicted solubility of these derivative inhibitors is close to that of MP3 (Figure S6), and although the double mutations increase the binding with RBD, their inhibitors are relatively less soluble than MP3 or LCB1 but they are still soluble (Figure S6). The MP15 inhibitor may display higher permeability and potency than MP3 due to the presence of one extra hydrophobic residue. MP15 also exhibits higher thermostability profile with T_m_ equal to 96 ± 2.9 °C (Figure S7).

To further verify that the increase in binding comes from the substituted AAs, the key interacting AAs in M15-MD (E11V + D17R) are analyzed as shown in Figure S8c,d. The stronger binding is primarily due to the D17R mutation, which provides a −4.15 kcal/mol energy boost. On the other hand, the E11V mutation gains an energy increase of only −0.73 kcal/mol at a local position in addition to its impact on the nearest neighbor AA (Y^10^) to get an extra −1.2 kcal/mol. Interestingly, MP15 interacts with a larger number of AAs on RBD comparing to MP3 and its total contribution to BFE is 51% or 1% larger than MP3.

## 3. Discussion

### 3.1. DFT Results

We have carried out the DFT calculations of five models to complement the detailed results from the MD simulations in Section 2. They are listed in Table 2 as M1(a)-DFT, M1(b)-DFT, M2-DFT, M3-DFT, and M15-DFT. These models are truncated in size without compromising the essential characteristic at the interfaces of the complexes to provide the atomistic details of the interaction based on rigorous quantum chemical calculations. The results will focus on the partial charge distribution, interatomic bonding between all pairs of atoms, and the hydrogen-bonding network.

**Figure 3:**
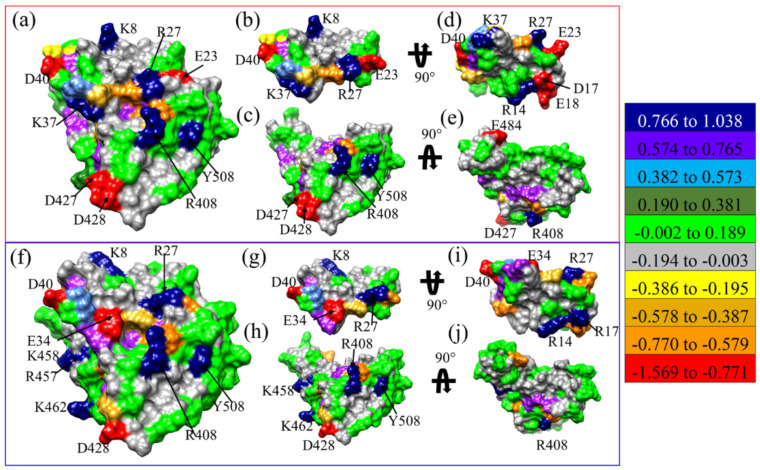
PC on the solvent excluded surface for (**a**) M3-DFT model (**b**,**c**) shows separated RBD with MP3, and (**d**,**e**) shows the rotated surface of RBD and MP3. Surface PC in the (**f**) M15-DFT model (**g**,**h**) shows separated RBD with MP15, and (**i**,**j**) shows rotated RBD and MP15. The color bar shows the total PC for different AAs from red (negative) to blue (positive). The navy blue and red AAs are identified and marked.

The partial charge (PC) on each AAs for M1(a)-DFT, M1(b)-DFT, M2-DFT, M3-DFT, and M15-DFT are shown in Figures S9–S13, respectively, and PC of AAs on the solvent excluded surface of M3-DFT and M15-DFT are shown in Figure 3a–j, respectively. Highly positive and highly negative AAs of M3-DFT shown in Figure 3a are K^8^, R^27^, K^37^, R^408^, and Y^508^, and E^23^, D^40^, D^427^, and D^428^, respectively. Similarly, highly positive and highly negative AAs of M15-DFT in Figure 3f are K^8^, R^27^, R^408^, R^457^, K^458^, K^462^ and Y^508^, and E^34^, D^40^, and D^428^, respectively. The interacting surface of MPs shown in Figure 3d,i for M3-DFT and M15-DFT has AAs with more largely positive or negative PC than in RBD. The E^11^ and D^17^ of MP3 in M3-DFT is substituted to V^11^ and R^17^ of MP15 in M15-DFT. The highly negative PC of D^17^ in M3-DFT has flipped to highly positive PC of R^17^ in M15-DFT. D17R substitution is further discussed in Section 3.2.

**Figure 4:**
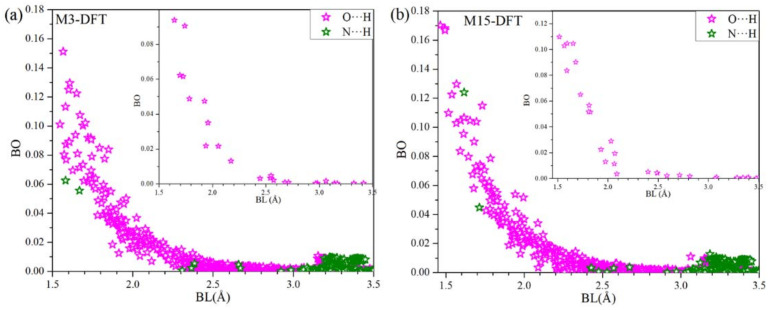
Hydrogen bonding distribution for the (**a**) M3-DFT and (**b**) M15-DFT. Inset: HBs between the respective RBD and miniprotein.

The bond order (BO) vs. bond length (BL) showing hydrogen bonding (HB) for M3-DFT and M15-DFT are shown in Figure 4a,b, respectively. There are significantly large number of O⋯H HBs than N⋯H HBs. Most of the HBs becomes very weak at around 2.5 Å. However, there are N⋯H HBs, which gets slightly higher from 3.2 Å to 3.5 Å. The inset shows HBs between the MP3:RBD and MP15:RBD in Figure 4a,b, respectively, with both consisting of only O⋯H HBs. Even though there are HBs with lower BO we believe their large number plays significant role in the interaction.

### 3.2. Combination of MD and DFT Results

The combination of comprehensive classical MD simulations and highly accurate DFT calculations is a promising technique for studying various biomolecular processes that have recently gained attention [42,43,44]. Each method has its unique features but also obvious limitations. By intelligently combining them, some of these limitations can be overcome and new, previously absent insights can be explicitly revealed [30]. For instance, the major drawbacks of using force field in MD simulation are the fixed partial charges (PCs) and the inability to describe forming or breaking of the covalent bonding between atoms during the chemical reaction [45]. One technique for mitigating these drawbacks is to accurately calculate PCs based on ab initio simulation and feed them into the MD force field. As in our previous study [30], the accurate PCs from DFT (Section 3.1) can be used for estimating the electrostatic interaction which may be a very important parameter to improve the accuracy of the MD force field. The rigorous interatomic bonding and PCs distribution on each atom and residue obviously complement what the MD simulations lack. On the other hand, the simulating all-atoms of the system on a long timescale (nanoseconds or longer) using ab initio methodologies is still a challenging task [46]. Therefore, it is necessary to resort to a classical MD for simulation all-atoms across a longer timescale and combine with ab initio calculations for a smaller section of the system containing all essential interacting AAs.

**Figure 5:**
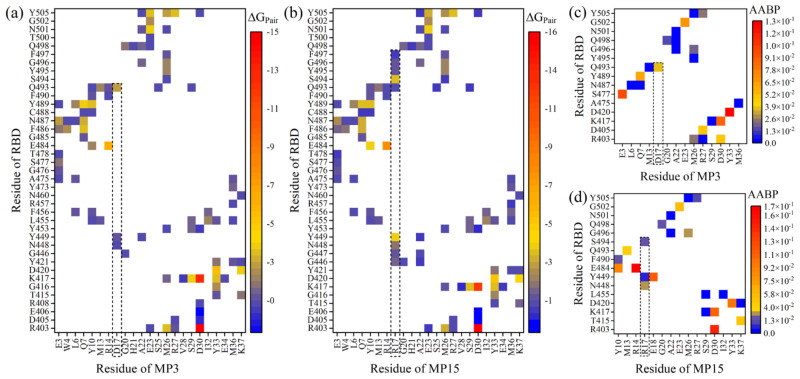
AA–AA interaction pair map between miniprotein and RBD of SARS-CoV-2 based on MD (**a**,**b**) and DFT (**c**,**d**) analysis. (**a**,**b**) AA–AA interacting pairs using pairwise BFE decomposition scheme for M3-MD (MP3:RBD) and M15-MD (MP15:RBD) models, respectively. (**c**,**d**) The AA–AA bond pair (AABP) for M3-DFT and M15-DFT models. Each square cell represents the intersection AA from RBD on the vertical axis and AA from miniprotein on the horizontal axis. These pairs have different strengths based on ΔG_Pair_ or AABP values.

Here is an example to investigate the nature of the AA–AA interacting pairs between the MP3 or MP15 and RBD using these two fundamentally different methodologies. Figure 5a,b show the AA–AA pair network maps of MP3:RBD and MP15:RBD complexes using the pairwise BFE decomposition scheme of the MM-GBSA method [30].

Quantitatively, the number of pairings in MP15:RBD is more than MP3:RBD (98 vs. 92 pairs). The substitution of D17 with R gains six new pairings with RBD in which the S^494^:R^17^ pair is the strongest one with ΔG_Pair_ of −3.14 kcal/mol (see the dash lines in Figure 5a,b). Additionally, some pairings between AAs of RBD and R^17^ are stronger than the same pairing with D^17^. For instance, Y^449^:R^17^, N^448^:R^17^, and G^446^:R^17^ have ΔG_Pair_ of −3.48, −1.41, and −1.21 kcal/mol, respectively, while the ΔG_Pair_ of Y^449^:D^17^, N^448^:D^17^ and G^446^:D^17^ are only −0.74, −0.45, and −0.15 kcal/mol. In contrast, the Q^493^:R^17^ is less strong than Q^493^:D^17^. All these results further support our conclusion that the D17R mutation is enhancing the binding affinity of miniprotein with RBD.

In Figure 5a,b, D^30^ of each miniprotein forms very strong ionic pairs with R^403^ and K^417^ of RBD with ΔG_Pair_ of ~−15.5 and −12.5 kcal/mol, respectively. E^484^:R^14^ and D^420^:Y^33^ are also strong with ΔG_Pair_ < −5 kcal/mol. There are 39 (35) pairs in MP15:RBD (MP3:RBD) has ΔG_Pair_ > −4 but ≤ −1 kcal/mol. The remaining pairs have ΔG_Pair_ > −1.

Figure 5d display the AA–AA bond pair (AABP) of corresponding DFT models (M3-DFT and M15-DFT). The AABP is a new concept to describe the interacting pair between two AAs and quantify their strengths based on ab initio calculations [47]. It includes both covalent bonds and HBs. The main observations from these figures can be summarized as follows: First, replacing D^17^ with R results in more contacts with RBD, supporting the MD findings. Second, D^30^ forms strong pairs with R^403^ and K^417^ and this is again confirmed by the MD analysis. Third, D^420^:Y^33^ is strongly binding in both models with AABP values of 0.1291 e and 0.1029 e in M3-DFT and M15-DFT, respectively, while the strong E^484^:R^14^ pair is only formed in M15-DFT with AABP value of 0.1724 e. Finally, even though both MP15:RBD and MP3:RBD has the same number of pairs based on AABP analysis (23 pairs), they have different AABP strengths and characteristics. AABP in MP15:RBD is stronger than MP3:RBD with overall AAPBs equal to 1.0248 e vs. 0.6641 e, respectively. This is clear evidence that both methods reach the same conclusion regarding the binding of MP15, viz. that it is being improved when D^17^ is replaced with R. The AABP for DFT models M1(a)-DFT, M1(b)-DFT, and M2-DFT are shown in Figure S14.

### 3.3. Potential Connection to Experimental Verifications

It is important to look out for a possible connection to experimental procedure to be reasonable. Solid-phase peptide synthesis (SPSS) will be used to synthesize the miniproteins (MP3-MP15, 40 amino acids) using a PurePep Chorus peptide synthesizer (Gyros Protein Technologies, Tucson, AZ, USA). The binding affinity of these miniproteins to SARS-CoV-2 RBD will be evaluated using surface plasmon resonance (SPR), which is a standard technique to study protein-protein interactions. Briefly, SARS-CoV-2 RBD protein will be used to coat a CM5 dextran sensor chip (Biosensing Instrument, Tempe, AZ) as we described before [48]. A series of concentrations of each miniprotein (0.1, 1, 10, 50, 100, 200, 500, and 1000 nM) will be analyzed to calculate the equilibrium dissociation constant (K_D_) values. Next, we will select the miniproteins that exhibit high affinity to SARS-CoV-2 RBD and study whether they can block the SARS-CoV-2 RBD/ACE2 interaction as we reported [35]. A 96-well plate will be coated with SARS-CoV-2 RBD protein, blocked with bovine serum albumin (BSA), and incubated with the miniproteins at room temperature for 1 h. After washing, biotinylated-ACE2 protein will be added to the wells and incubated for 1 h. Streptavidin-HRP and substrate will be finally added to measure absorbance at 450 nm. We will also evaluate whether the miniproteins can block the infection of a Spike (SARS-CoV-2) Pseudotyped Lentivirus with luciferase reporter (BPS Bioscience, San Diego, CA) in Vero-E6 cells. Briefly, Vero-E6 cells will be cultured in 96-well plates and then incubated with the pseudotyped virus alone or with the miniproteins at 37 °C for 1 h. The medium will be replaced with a refresh medium and incubated for 48 h. The cells will be harvested to measure luminescence and calculate the infection efficiency. These affinity and blocking assays will provide vital information about the accuracy of the modeling.

## 4. Materials and Methods

### 4.1. Model Construction

We explicitly design and simulate many minibinder:RBD models to find optimal MP with ultra-high binding affinity and understand its binding mode with RBD SARS-CoV-2. The initial structure of the RBD is obtained by removing the ACE2 receptor and all other molecules from the crystal structure of RBD:ACE2 (PDB ID: 6M0J) [31]. The original MPs, LCB1 and LCB3, are taken from Supplementary Materials of [20]. Two points must be mentioned here. First, the structures of LCB1:RBD and LCB3:RBD are also available from Supplementary Materials of [20] and their RBDs were also obtained from the 6M0J structure that we use. We do not use them, however, because we discovered that the cysteine residues in these complexes were not protonated. Second, the current study is initiated before LCB1:RBD and LCB3:RBD complexes are deposited in PBD with IDs 7JZU and 7JZM, respectively [20].

**Figure 6:**
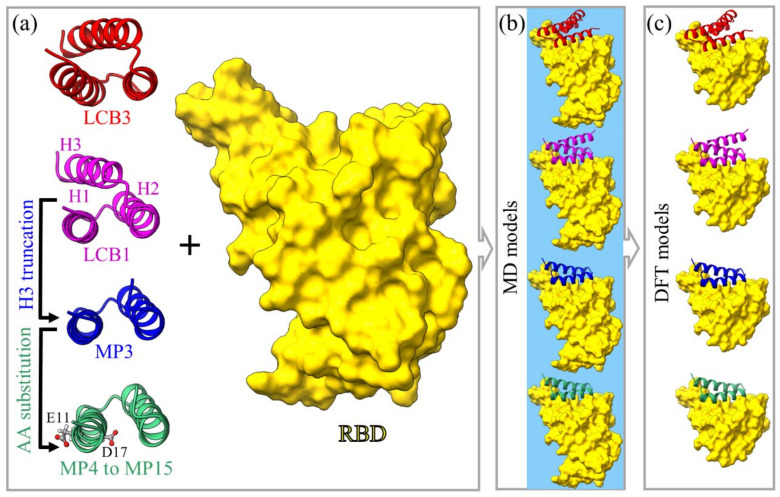
Graphical illustration of models for miniprotein candidates in ribbon form to target RBD SARS-CoV-2. (**a**) Both LCB3 and LCB1 have three alpha-helices (H1, H2, and H3). The H3 of LCB1 is truncated to generate MP3 to reduce its size followed by AA substitutions at its residues 11 and 17. For AA substitutions, we created 10 different models for point mutations and two models for double mutation (see Table 1). (**b**) The solvated models of the bound miniprotein:RBD complex for MD (water represented by blue background). (**c**) The smaller DFT models (without water) built from MD models in (**b**) for MPs and small portion of RBD including only residues 401 to 508. For better visual clarity, the salt ions are hidden in (**b**,**c**).

We now outline the procedures for building the models as follows:

i. The initial models for LCB1:RBD and LCB3:RBD complexes are similar to [20] but without an extra AA in RBD (195 vs. 194 AAs of 6M0J) and with protonated cysteine residues. The structure comparison tool in the UCSF Chimera software [49] is used to align the RBD in 6M0J with LCB1:RBD or LCB3:RBD of [20]. The RBDs are then replaced by the ones from the 6M0J.
ii. Explicit H atoms are added to the saved structure using LEaP module in AMBER [26].
iii. Each complex is solvated with 10,000 water molecules with appropriate ions numbers using the TIP3P explicit water model implemented in AMBER [26,50]. The most recommended AMBER force field ff14SB is used to represent the parameterizations [51].
iv. The same procedures are adopted to generate the new design for the other minibinder:RBD complex. The details of these constructions are illustrated in Figure 6, Table 2, and Table S2 in Supplementary Materials.

We now start the discussion of the 15 simulation models in the present study listed in Table 2. We start with M1 and M2.

#### 4.1.1. Models M1 and M2

Cao et al. have employed two computational approaches to design MPs: one was based on scaffolds built around the alpha-helix of the ACE2 receptor; the second was to design synthetic MPs completely from scratch and independent of known RBD-binding interactions [20]. The latter approach produced eight highest-affinity MPs known as LCB1 to LCB8. Among them, LCB1 and LCB3 are the most potent synthetic antivirals that bind to RBD with a dissociation constant (K_D_) of less than 1 nM, which is too strong to be measured accurately with the experimental biolayer interferometry (BLI) technique used [20].

In this regard, the MM-GBSA method based on MD is used to calculate their BFE and elucidate their thermodynamics and binding mechanisms at the microscopic level. We created two MD simulation models, M1-MD and M2-MD, for the LCB3:RBD and LCB1:RBD complexes, respectively. Their contents are described in Table S2.

Our other main goal is to explore the interatomic interactions of minibinder:RBD at the atomic scale using ab initio approach. However, ab initio calculations of such large models are currently impossible. So, we shrunk the M1-MD and M2-MD down to a much more manageable size containing only the most relevant AAs at their interface. From M1-MD, we constructed two new DFT models named as M1(a)-DFT and M1(b)-DFT. M1(a)-DFT is taken from the initial structure before MD simulation while the second one is created from the last step of MD. The reason for constructing two M1-DFT models is to accommodate the conformational change in RBD during the MD simulation (Figure S15). M1-DFT models contain all 64 AAs of LCB3, the segment of RBD, 5 Na^+^ ions to neutralize the total charge, and without water. The segment of RBD contains 108 AAs (residue 401–508), including all residues of the receptor-binding motif (RBM) (residues 438–508) and extra RBD residues (401–437). The selection of this segment is based on the key AAs that interact between LCB3 and RBD (see Section 2). The 2 DFT models are fully optimized using VASP (see Method section in Supplementary Materials). In the same manner, we generated one M2-DFT model from the last step of M2-MD. It contains all AAs of LCB1 (56 AAs), the same segment of RBD used in M1-DFT (108 AAs), and 7 Na^+^ ions to neutralize the model.

#### 4.1.2. Model M3

The design of small-sized MPs such as MP3 is one of the main goals of this study. As showed in Section 2, the alpha-helix 3 (H3) of LCB1 has no significant contribution to BFE. This observation leads us to design MP like LCB1 but without H3 and label it as MP3 which contains only 40 AAs. One main advantage of MP3 is its small size, which results in lower production costs and high yield. This smaller size may also lead to high selectivity binding to a target and low interference with biological processes, thus reducing its side effects [25,35]. The structure of the last step in M3-MD is used to generate M3-DFT which has all 40 AAs of MP3, a similar segment of 108 AAs in RBD, and 3 Na^+^ ions.

#### 4.1.3. Models M4 to M15

To enhance the binding properties of MP3 to RBD, we generate additional MD models M4 to M15. These involve single and double mutations at certain positions in MP3 in which the residues E11 and/or D17 are substituted by other selective AAs. Residue E11 does not face the RBD of the S-protein and is classified as acidic AA. We replace it by the following AAs one at a time: D (acidic AA), R (basic AA), T (hydroxylic polar AA), M (sulfuric nonpolar AA), Q (amide polar AA) and V (aliphatic nonpolar AA). These substitutions in MP3 render E11 residue to mutate into different AAs by adopting the Dunbrack backbone-dependent rotamer library [52] implemented by UCSF Chimera [53]. For each substitution, we create only one MD model and labeled them as M4-MD to M9-MD, where their corresponding MPs are MP4 to MP9. Since the substitution of E11 residue with all 20 canonical AAs is computationally time-consuming, we limited them to a few AAs based on the site saturation mutagenesis (SSM) analyses of LCB1 [20]. Similarly, the D17 residue is substituted by residues E, R, T, and M. Their corresponding MD models are labeled as M10-MD to M13-MD and their MPs are MP10 to MP13. Here, D17 is facing the RBD so their alterations may promote the thermodynamic binding profile of MP3. Another proposed alteration is the double mutations at residues E11 and D17, we generate two such models and labeled them as M14-MD and M15-MD (their MPs are MP14 and MP15). These double mutation models are constructed based on the best thermodynamic binding profile from the point mutations at E11 to Q11 or V11 and D17 to R17. Lastly, we created one DFT model from the last step of M15-MD and labeled it as M15-DFT. M15-DFT has all 40 AAs of MP15 and 108 AAs of the RBD segment without water molecules or ions.

### 4.2. Methods

Two methodologies have been adopted to investigate the binding phenomenology of fifteen MPs with RBD SARS-CoV-2 at the molecular, amino acid, and atomic levels. The first approach is to use classical molecular dynamics to understand the dynamic and binding properties of the minibinder:RBD complex. The entire binding affinity profiles of the complexes have been computed using the Molecular Mechanics-Generalized Born Surface Area (MM-GBSA) method [30,54,55,56,57]. Additionally, the per-residue and pairwise BFE decompositions have been applied to study the role of the structural modification on LCB1 binding and the impact of selected mutations at certain positions on MP3 binding.

Drug discovery and development is inherently a time-consuming and expensive process, with a high failure rate of 90% of drugs entering clinical trials failing to get FDA approval and reaching the market [58]. Although high-throughput screening (HTS) experiments are the fastest approach, they are still expensive and require many targets and ligands. Therefore, computer-aided drug discovery (CADD) technologies are alternatively used to reduce the number of ligands that need to be screened in HTS assays in order to fast the drug discovery process [58]. Structure-based (SB) and ligand-based (LB) drug discovery are the two primary categories of CADD approaches. MD simulation and the MM-GBSA method have been widely used in the drug design, particularly in the early stages of SB virtual screening [55,59,60,61,62,63]. They are used to gain insight into not only how ligands bind to target proteins, but also the pathways of interaction and to account for target flexibility. The most well-known examples of how MD simulations have contributed to the development FDA-approved drugs are Raltegravir, a HIV integrase inhibitor [64,65] and Zanamivir, a neuraminidase inhibitor against influenza A and B virus [66]. More details are fully described in references [55,58,59,60,61,62,63,64,65,66].

The second approach relies on more accurate ab initio quantum chemical calculations based on density functional theory (DFT). The structures for each DFT are optimization first by using Vienna ab initio simulation package (VASP) [27,28]. This is followed by using a different DFT method developed in-house, the all-electron orthogonalized linear combination of atomic orbitals (OLCAO) method [29]. OLCAO method is extremely efficient in probing the electronic structure, partial charge distributions, and interatomic bonding including the hydrogen bonding network and the AA–AA interactions [30,47,67,68,69,70,71]. These methods are fully described in Supplementary Materials.

## 5. Conclusions

Designing and developing high-affinity miniproteins to stall S-protein SARS-CoV-2 entry is a promising therapeutic scheme for COVID-19 treatment. Based on the most promising synthetic miniprotein LCB1 developed by the Baker group [20], many minproteins have been engineered and simulated to target RBD SARS-CoV-2 with goal to optimize a new design of the miniprotein that enables to compete ACE2 binding. To achieve that, the LCB1 is subjected to two modifications: structural modification to reduce its size, followed by single and double amino acid substitution at specific positions to enhance its binding affinity. Our methodologies are based on traditional MD simulations and ab initio DFT calculations. This work reveals the ability of computational models to respond to SARS-CoV-2 or other future viral threats. Our investigation yields the following solid conclusions:

i. From detailed and systematic MD simulations at the microsecond time scale, the complete energetic profile and interaction spectrum of the miniprotein:RBD complexes have been obtained, suggesting that either the original miniproteins (LCB3 or LCB1) or the designed ones, obtained from the LCB1, can compete for ACE2 binding due to their high binding affinity with RBD and selectivity to occupy the binding site of ACE2 on RBD.
ii. Truncation of the alpha-helix 3 (H3) of LCB1 results in the development of a small candidate (MP3) with a better binding profile to RBD. Additionally, amino acid substitutions at residues 11 and 17 of MP3 enhance its binding more, especially D17R. The D17R substitution shows significant change in PC, which could be the reason behind their enhanced binding.
iii. Since this work is limited to only the RBD of wild-type SARS-CoV-2, we plan to investigate the ability of the best candidate from this study (MP15) to inhibit the RBD of existing SARS-CoV-2 variants, particularly Omicron RBD.
iv. Because of the computational demands, amino acid substitutions of MP3 are restricted to a few AAs at only two sites (11 and 17), which do not cover every residue of MP3 and potential substituted of the 20 AAs at each one.

## Figures and Tables

**Table 1:** Predicted BFE (kcal·mol^−1^) and its thermodynamic components at 0.15 M salt of M1-MD (LCB3:RBD) and M2-MD (LCB1:RBD) models. The last column represents differences in energy components between the two models. SEM in parentheses is the standard error of the mean.

Energy	M1-MD (SEM)	M2-MD (SEM)	ΔΔG_(M2-M1)_
ΔE_vdW_	−93.26 (0.13)	−91.32 (0.10)	1.95
ΔE_ele_	−492.67 (0.62)	−516.55 (0.58)	−23.88
ΔE_MM_	−585.93 (0.66)	−607.86 (0.59)	−21.93
ΔG_GB_	527.00 (0.57)	540.09 (0.55)	13.09
ΔG_SA_	−14.36 (0.02)	−13.86 (0.01)	0.50
ΔG_sol_	512.63 (0.57)	526.22 (0.55)	13.59
ΔG_ele_ ^1^	34.32 (0.142)	23.54 (0.12)	−10.78
−TΔS	−47.82 (1.50)	−52.91 (1.05)	−5.09
ΔG_bind_	−25.48 (0.60)	−28.73 (0.51)	−3.25

^1^ ΔG_ele_ = ΔE_ele_ + ΔG_GB_.

**Table 2:** The constructed models for MD simulations and DFT calculations (**bold black**) of the miniprotein (MP) and RBD complex. The original H3 sequence in LCB1 is underlined and truncated to create MP3. All models (M3 to M15) are generated based on MP3 sequence. The residues 11 and 17 of MP3 (marked in bold **red**) are altered to other selective AAs (marked in bold **blue**).

Model ^1^	Total Atoms Number	MP	MP Sequence
M1-MD	34099	MP1 (LCB3)	NDDELHMLMTDLVYEALHFAKDEEIKKRVFQLFELADKAYKNNDRQKLEKVVEELKELLERLLS
**M1(a)-DFT**	**2773**		
**M1(b)-DFT**	**2773**		
M2-MD	33961	MP2 (LCB1)	DKEWILQKIYEIMRLLDELGHAEASMRVSDLIYEFMKKGD ERLLEEAERLLEEVER
**M2-DFT**	**2635**		
M3-MD	33678	MP3	DKEWILQKIY**E**IMRLL**D**ELGHAEASMRVSDLIYEFMKKGD
**M3-DFT**	**2352**		
M4-MD	33675	MP4	DKEWILQKIY**E**IMRLL**D**ELGHAEASMRVSDLIYEFMKKGD
M5-MD	33687	MP5	DKEWILQKIY**R**IMRLL**D**ELGHAEASMRVSDLIYEFMKKGD
M6-MD	33676	MP6	DKEWILQKIY**D**IMRLL**D**ELGHAEASMRVSDLIYEFMKKGD
M7-MD	33679	MP7	DKEWILQKIY**R**IMRLL**D**ELGHAEASMRVSDLIYEFMKKGD
M8-MD	33679	MP8	DKEWILQKIY**T**IMRLL**D**ELGHAEASMRVSDLIYEFMKKGD
M9-MD	33678	MP9	DKEWILQKIY**M**IMRLL**D**ELGHAEASMRVSDLIYEFMKKGD
M10-MD	33681	MP10	DKEWILQKIY**Q**IMRLL**D**ELGHAEASMRVSDLIYEFMKKGD
M11-MD	33690	MP11	DKEWILQKIY**V**IMRLL**D**ELGHAEASMRVSDLIYEFMKKGD
M12-MD	33679	MP12	DKEWILQKIY**E**IMRLL**E**ELGHAEASMRVSDLIYEFMKKGD
M13-MD	33682	MP13	DKEWILQKIY**E**IMRLL**R**ELGHAEASMRVSDLIYEFMKKGD
M14-MD	33693	MP14	DKEWILQKIY**E**IMRLL**T**ELGHAEASMRVSDLIYEFMKKGD
M15-MD	33692	MP15	DKEWILQKIY**E**IMRLL**M**ELGHAEASMRVSDLIYEFMKKGD
**M15-DFT**	**2362**		

^1^ The labels -MD and -DFT after the model number indicate which method is used for simulation.

## Data Availability

All data are listed in tables or presented in figures in main text or Supplementary Materials.

## Supplementary Materials

The following are available online at https://www.mdpi.com/article/10.3390/ijms23020838/s1.
