# Supplementary material for: Computational Design of Miniproteins as SARS-CoV-2 Therapeutic Inhibitors
Source: Int J Mol Sci. 2022 Jan 13;23(2):838. doi: 10.3390/ijms23020838 (PMC8776159; doi:10.3390/ijms23020838)
Supplement: Supplementary file 1 [file ijms-23-00838-s001.zip › ijms-1541918-supplementary-done.pdf]

Supplementary Materials

Computational Design of Miniproteins as SARS-CoV-2 Therapeutic Inhibitors

Bahaa Jawad [1,2], Puja Adhikari [1], Kun Cheng [3], Rudolf Podgornik [4,5,6] and Wai-Yim Ching [1,*]

1 Department of Physics and Astronomy, University of Missouri-Kansas City, Kansas City, MO 64110, USA; bajrmd@mail.umkc.edu (B.J.); paz67@umkc.edu (P.A.)
2 Department of Applied Sciences, University of Technology, Baghdad 10066, Iraq
3 Division of Pharmacology and Pharmaceutical Sciences, School of Pharmacy, University of Missouri-Kansas City, Kansas City, MO 64108, USA; chengkun@umkc.edu
4 Wenzhou Institute of the University of Chinese Academy of Sciences, Wenzhou 325000, China; rudolf.podgornik@fmf.uni-lj.si
5 School of Physical Sciences and Kavli Institute of Theoretical Science, University of Chinese Academy of Sciences, Beijing 100049, China
6 CAS Key Laboratory of Soft Matter Physics, Institute of Physics, Chinese Academy of Sciences, Beijing 100090, China
* Correspondence: Chingw@umkc.edu

1. Extended Methods

1.1. Molecular Dynamic (MD) Simulation

All-atom explicit solvent molecular dynamics (MD) simulations have been performed of minibinder:RBD models by using the AMBER 18 simulation package on a microsecond time-scale. Standard MD simulation procedures: minimization, heating, equilibration, and MD production are used as follows. First, each model is minimized in six successive stages using 10,000 steps of minimization (5000 steps of steepest descent and followed by 5000 steps of a conjugate gradient). These minimization stages are implemented to remove bad-overlap contacts and enable the system to adjust to the chosen force fields. In the first five stages, different restraint force constants of 500, 250, 100, 10, 1 kcal/mol-Å^2 were applied to hold the solute (MP:RBD complex) in a fixed position and optimize the positions of water molecules and ions. In the final stage, all-atoms in the model are minimized without any constraints. Second, each model is heated by gradually raising the temperature from 0 K to 310 K for 310 picoseconds (ps) using an NVT ensemble with a weak restraint of 10 kcal/mol-Å^2 on the solute. Subsequently, models are equilibrated for 5 ns without constraint to achieve the necessary density using an NPT ensemble at a constant pressure of 1 bar and a temperature of 310 K. Finally, the NPT production dynamics are performed for 1000 ns (1 μs) in all models at constant pressure (1 bar) and temperature (310 K). Rather than run a continuously single MD simulation from 0 to 1μs, we performed 10 successive MD simulations one after the other using the structure file from the previous run as the input for the next run. The length of each MD run is 100 ns, 1000 ns in total (10 x 100 ns). The atomic coordinates from MD trajectories were saved every 2 ps for subsequent binding free energy (BFE) analysis. In equilibration and production procedures, the following settings are used: Langevin dynamics to control temperature, 2 ps as the pressure relaxation time, the SHAKE algorithm to constrain the motion of hydrogen-containing bonds with a 2 fs time step [1], and the Particle Mesh Ewald (PME) method to address long-range electrostatic interactions [2]. Both the direct space PME and the Lennard-Jones cut-offs are set at 10 Å. All these procedures are done using the PMEMD.CUDA module in AMBER [3,4].

1.2. Binding Free Energy (BFE) Calculations

Molecular mechanics generalized Born surface area (MM-GBSA) method is used to calculate the BFE between the miniprotein (MP) and RBD SARS-CoV-2. This is an end-point BFE method that combines the molecular mechanics (MM) with an implicit GBSA

continuum solvent model [5,6]. The single-trajectory protocol (STP) of MM-GBSA method is adopted in our BFE calculations. In STP approach, all ensembles can be extracted from a single MD simulation of the bound MP:RBD complex by only simulating the complex and obtaining the average ensemble of the free receptor (RBD) and ligand (MP). BFE is determined as the difference between the free energies of the bound state of MP:RBD complex ($G_{COM, sol}$) and the unbound state of RBD ($G_{RBD, sol}$) and MP ($G_{MP, sol}$) [5–8].

$$\Delta G_{bind} = G_{(COM,sol)} - G_{(RBD,sol)} - G_{(MP,sol)} \quad (1)$$

Each term in Eq. (1) can be computed from contributions of gas-phase MM energy, solvent-free energy (G_{sol}), and the conformational entropy (-TS) and expressed as

$$G = E_{MM} + G_{sol} - TS \quad (2)$$

Thus Equation (1) can be written as

$$\Delta G_{bind} = \Delta E_{MM} + \Delta G_{sol} - T\Delta S = \Delta G_{vac} + \Delta G_{sol} \quad (3)$$

$$\Delta E_{MM} = \Delta E_{int} + \Delta E_{ele} + \Delta E_{vdW} \quad (4)$$

$$\Delta G_{sol} = \Delta G_{GB} + \Delta G_{SA} \quad (5)$$

$$\Delta G_{SA} = \gamma . SASA + b \quad (6)$$

$$\Delta G_{vac} = \Delta E_{MM} - T\Delta S \quad (7)$$

ΔE_{MM} includes the changes in the bonded (ΔE_{int}), the non-bonded electrostatic (ΔE_{ele}) and the van der Waals (ΔE_{vdW}) energies. ΔG_{sol} is partitioned into an electrostatic or polar solvation energy part (ΔG_{GB}), and a non-electrostatic or nonpolar part (ΔG_{SA}) between the solute and the continuum solvent. The polar contribution $\Delta G_{GB/PB}$ is typically computed using either the generalized Born (GB) model as in our study, or the Poisson–Boltzmann (BP) model, whereas the nonpolar term (ΔG_{SA}) is estimated from a linear dependence on the solvent-accessible surface area (SASA) [5]. The free energy of the total electrostatic contribution is the sum of ΔE_{ele} and ΔG_{GB} ($\Delta G_{ele} = \Delta E_{ele} + \Delta G_{GB}$). The change in conformational entropy (-TΔS) contains three entropy terms: translational, rotational, and vibrational. The first two terms are estimated using the standard statistical mechanical formulas, while the vibrational entropy is approximated either *via* a normal-mode analysis of the vibrational frequencies as adopted in our calculations or *via* a quasi-harmonic approximation [5]. 10 snapshots along whole MD simulation have been used to calculate entropy Lastly, ΔG_{vac} is a contribution stemming from the solute.

Once the MD simulations are done, the snapshots are taken for every 100 ps over the whole 1 μs, so in total 5000 snapshots are extracted for the BFE post-process analysis. MMPBSA.py program in AMBER 18 is used to perform this BFE analysis depending on the MM-GBSA method with the following input file [5]. The model developed by Onufriev et al. (GB^{OBC} which GB=2) is applied as the GB model [9] and set mbondi2 radii are prepared. A surface tension coefficient (γ) of 0.0072 kcal/mol-Å^2 and zero correction constant (b=0) are employed to calculate ΔG_{SA}. The value of the exterior dielectric constant is set to 78.3 while the dielectric constant for the solute was set to its vacuum value, *i.e.*, 1. The salt concentration is set to 0.15 M. Besides that analysis, the *Per-residue* and *pairwise* BFE decompositions of the interaction between minibinders and RBD are also carried out by using the MMPBSA.py module.

1.3. Structural Relaxation Using VASP

The selected DFT models are all fully relaxed by using the Vienna ab initio simulation package (VASP) known for its efficiency in structure optimization [10]. The projector augmented wave (PAW) method with Perdew-Burke-Ernzerhof (PBE) exchange-correlation functional [11] within the generalized gradient approximation (GGA) are adopted. The following input parameters are used in VASP: energy cut-off 500 eV, electronic convergence of 10^{-4} eV, force convergence criteria for ionic steps at -10^{-2} eV/Å, and a single k-point sampling. All VASP relaxations were performed at the National Energy Research Scientific Computing (NERSC) facility at the Lawrence Berkeley Laboratory with special

allocations and at the Research Computing Support Services (RCSS) of the University of Missouri System. Due to the high accuracy required in structural relaxations and the slow convergence of large complex biomolecular systems, the computational resources used are quite large. The main aspect of geometrical optimization in VASP is to produce more realistic structures that will be used as input for further DFT calculations as described below.

1.4. Electronic structure and interatomic bonding using OLCAO

For the electronic structure and interatomic interactions of the MP:RBD DFT models, we use a very different DFT method with VASP-relaxed structures as input, the all-electron orthogonalized linear combination of atomic orbitals (OLCAO) method [12], developed in-house. The efficacy of using these two distinct DFT codes in many biomolecule systems has been well documented [13–15] including the many domains of S-protein SARS-CoV-2 and RBD:ACE2 interface complex [16–19]. The key feature of the OLCAO method is the provision for the effective charge (Q^*) on each atom and the bond order (BO) values $\rho_{\alpha\beta}$ between any pairs of atoms. They are obtained from the *ab initio* wave functions with atomic basis expansion:

$$Q_\alpha^* = \sum_i \sum_{m,occ} \sum_{j,\beta} C_{i\alpha}^{*m} C_{j\beta}^{m} S_{i\alpha,j\beta} \quad (8)$$

$$\rho_{\alpha\beta} = \sum_{m,occ} \sum_{i,j} C_{i\alpha}^{*m} C_{j\beta}^{m} S_{i\alpha,j\beta}. \quad (9)$$

In the above equations, $S_{i\alpha,j\beta}$ are the overlap integrals between the ith orbital in α^{th} atom and the jth orbital in the βth atom. $C_{j\beta}^{m}$ are the eigenvector coefficients of the m^{th}occupied molecular orbital level. The partial charge (PC) or ($\Delta Q_\alpha = Q_\alpha^0 - Q_\alpha^*$) is the deviation of the effective charge Q_α^* from the neutral atomic charge Q_α^0 on the same atom α. The BO quantifies the strength of the bond between two atoms and usually scales with the bond length (BL). The BL should be more accurately interpreted as the distance of separation of the two atoms since the BO value is influenced by the surrounding atoms. The calculation of PC and BO is based on the Mulliken scheme [20,21].

The BO $\rho_{\alpha\beta}$ in Eq. (9) is further extended to quantify the bonding strength between a pair of amino acids (u,v) called amino acid -amino acid bond pair (AABP) [18]. In many cases, the use of AABP is more useful than interatomic bonding between a pair of atoms for biomolecular systems

$$AABP(u,v) = \sum_{\alpha \epsilon u} \sum_{\beta \epsilon v} \rho_{\alpha i,\beta j}. \quad (10)$$

AABP considers all possible bonding between two amino acids including both covalent and hydrogen bonding (HB).

Table S1. Impact AAs substitution on BFE of MP3:RBD complex. The predicated BFE (kcal.mol^{-1}) and energetic components are calculated at 0.15 M salt by using the MM-GBSA method.

Model	Energy (kcal.mol^{-1})								
	ΔE_{vdW}	ΔE_{ele}	ΔE_{MM}	ΔG_{GB}	ΔG_{SA}	ΔG_{sol}	ΔG_{ele}	$-T\Delta S$	ΔG_{bind}
M3-MD	−91.64	−392.48	−484.12	413.14	−14.27	398.87	20.66	−55.06	−30.19
M4-MD	−91.87	−387.92	−479.79	409.22	−14.14	395.08	21.29	−54.51	−30.20
M5-MD	−91.27	−361.52	−452.79	381.48	−14.37	367.11	19.96	−55.57	−30.11
M6-MD	−90.91	−379.38	−470.29	398.26	−14.37	383.89	18.88	−55.36	−31.04
M7-MD	−91.97	−367.68	−459.65	389.20	−14.34	374.86	21.52	−54.52	−30.27
M8-MD	−92.22	−385.88	−478.10	405.95	−14.52	391.43	20.07	−55.50	−31.17
M9-MD	−91.88	−379.91	−471.79	398.41	−14.40	384.02	18.50	−55.90	−31.88
M10-MD	−93.55	−399.84	−493.38	420.96	−14.78	406.18	21.12	−55.77	−31.43
M11-MD	−99.59	−311.70	−411.29	334.18	−15.39	318.80	22.48	−56.94	−35.55
M12-MD	−93.43	−329.26	−422.70	353.28	−14.42	338.86	24.02	−55.26	−28.58
M13-MD	−96.58	−334.11	−430.69	358.08	−14.82	343.26	23.97	−54.93	−32.50
M14-MD	−96.96	−309.32	−406.28	329.96	−14.97	315.00	20.64	−55.84	−35.44
M15-MD	−98.23	−317.36	−415.59	336.37	−15.26	321.10	19.00	−57.12	−37.37

Table S2. Summary of MP:RBD complex models used in MD simulations and DFT calculations (bold black).

Model	Number Atoms in MP	Number atoms in RBD or RBD Segment	Number of Water Atoms	Number of Ions	Total number of Atoms
M1-MD	1095	3001	30000	3 Na^+	34099
**M1(a)-DFT**	**1095**	**1673**	**---**	**5 Na^+**	**2773**
**M1(b)-DFT**	**1095**	**1673**	**---**	**5 Na^+**	**2773**
M2-MD	955	3001	30000	5 Na^+	33961
**M2-DFT**	**955**	**1673**	**---**	**7 Na^+**	**2635**
M3-MD	676	3001	30000	1 Na^+	33678
**M3-DFT**	**676**	**1673**	**---**	**3 Na^+**	**2352**
M4-MD	673	3001	30000	1 Na^+	33675
M5-MD	685	3001	30000	1 Cl^-	33687
M6-MD	675	3001	30000	---	33676
M7-MD	678	3001	30000	---	33679
M8-MD	678	3001	30000	---	33679
M9-MD	677	3001	30000	---	33678
M10-MD	679	3001	30000	1 Na^+	33681
M11-MD	688	3001	30000	1 Cl^-	33690
M12-MD	678	3001	30000	---	33679
M13-MD	681	3001	30000	---	33682
M14-MD	690	3001	30000	2 Cl^-	33693
M15-MD	689	3001	30000	2 Cl^-	33692
**M15-DFT**	**689**	**1673**	**---**	**---**	**2362**

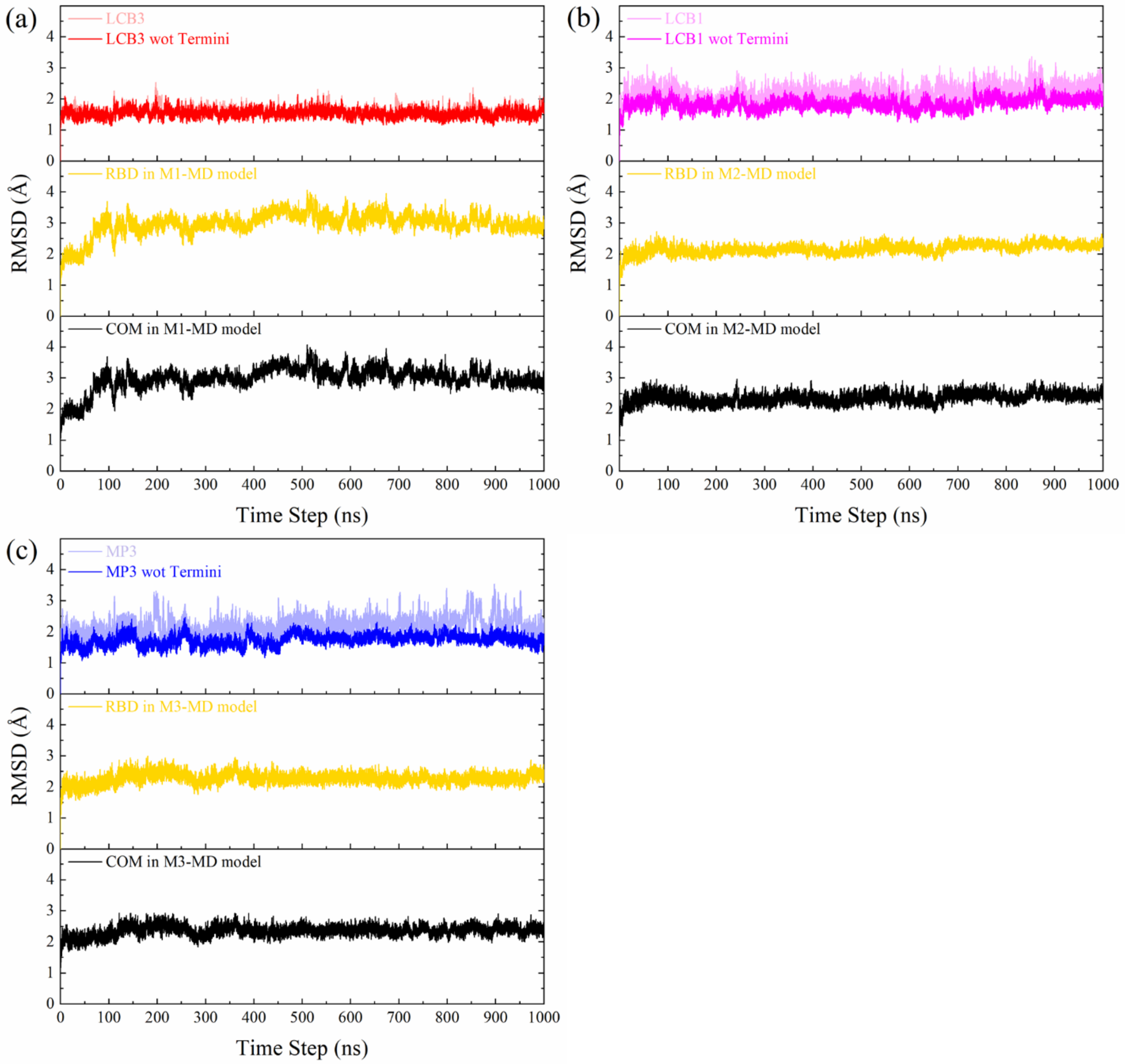

Figure S1. The root mean square deviation (RMSD) of the heavy atoms of MP:RBD SARS-CoV-2 complexes vs simulation time (ns). (**a**) For M1-MD; (**b**) for M2-MD, and (**c**) for M3-MD models. The average RMSDs of RBD in M1-MD, M2-MD, M3-MD, M15-MD are 2.97 ± 0.36 Å, 2.17 ± 0.16 Å, 2.27 ± 0.17 Å respectively, while their complexes have averaged values of 2.78 ± 0.3 Å, 2.35 ± 0.17 Å, and 2.36 ± 0.17 Å. Additionally, both LCB3, LCB1, and MP3 have achieved stability throughout the 1 μs long simulations as shown by smooth fluctuations with averaged RMSDs of 1.57 ± 0.15 Å, 2.2 ± 0.23 Å, and 2.2 ± 0.27 Å, respectively. When both termini of miniproteins are not included, these RMSDs are lowered to 1.53 ± 0.12 Å, 1.8 ± 0.18 Å and 1.73 ± 0.18 Å, indicating the relatively larger RMSD in LCB1 or MP3 are mainly arising from their termini.

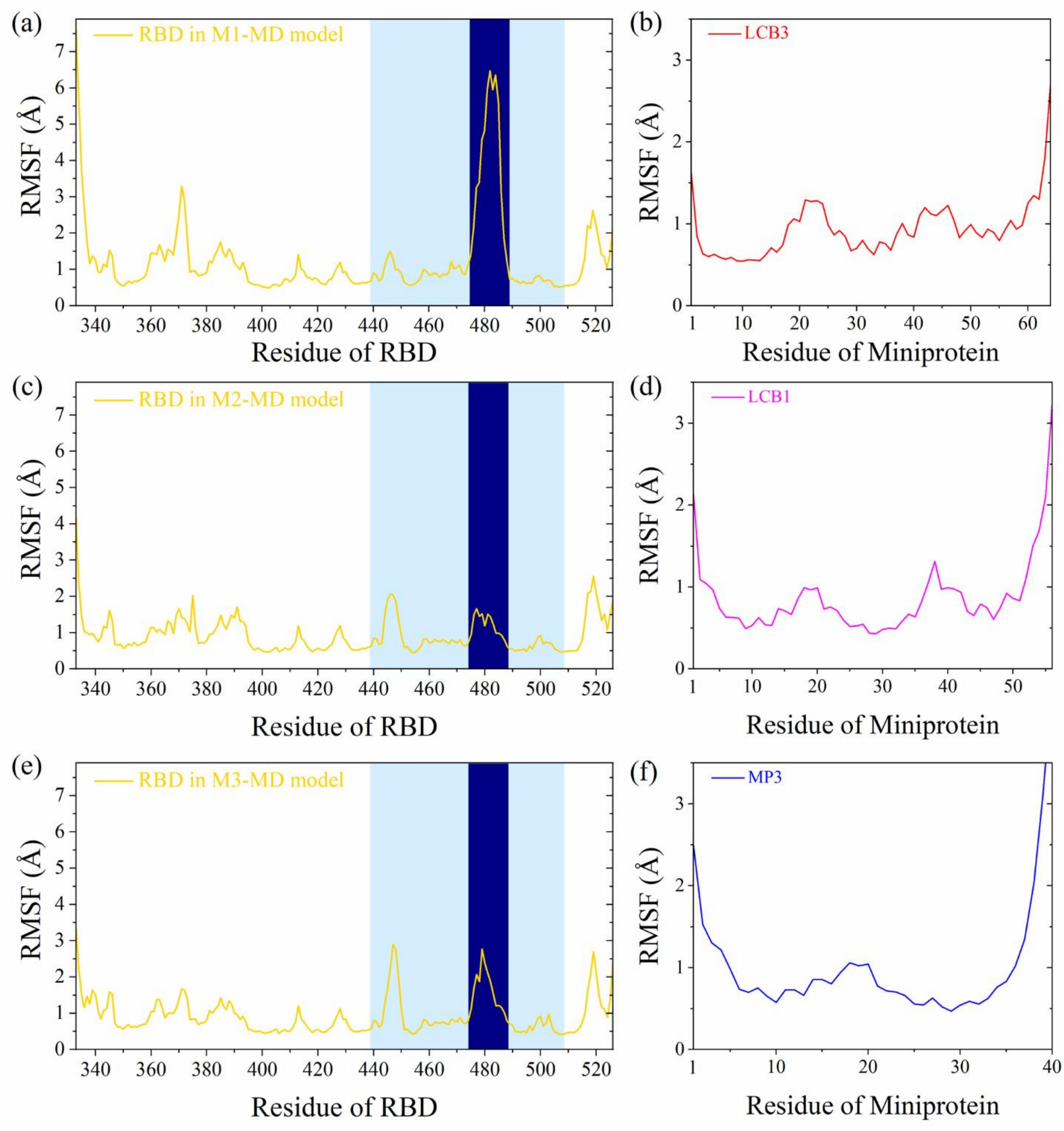

Figure S2. The root mean square fluctuation (RMSF) of the residues at MP:RBD complexes. The RBM and loop 3 of RBD are highlighted in light blue and navy blue. (**a**,**b**) for M1-MD; (**c**,**d**) for M2-MD, and (**e**,**f**) for M3-MD models.

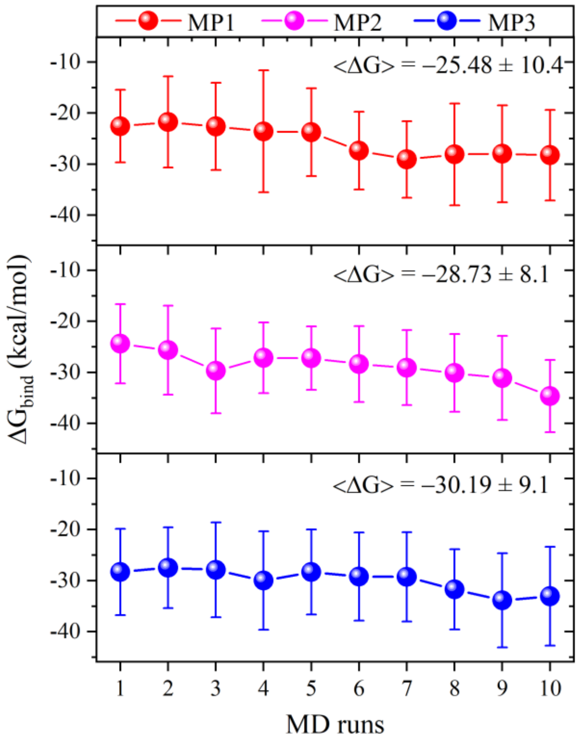

Figure S3. Convergence plot of the calculated binding free energy (ΔG_{bind}) as a function of the 10 successive MD. The length of each MD run is 100 ns, 1000 ns in total (10 × 100 ns).

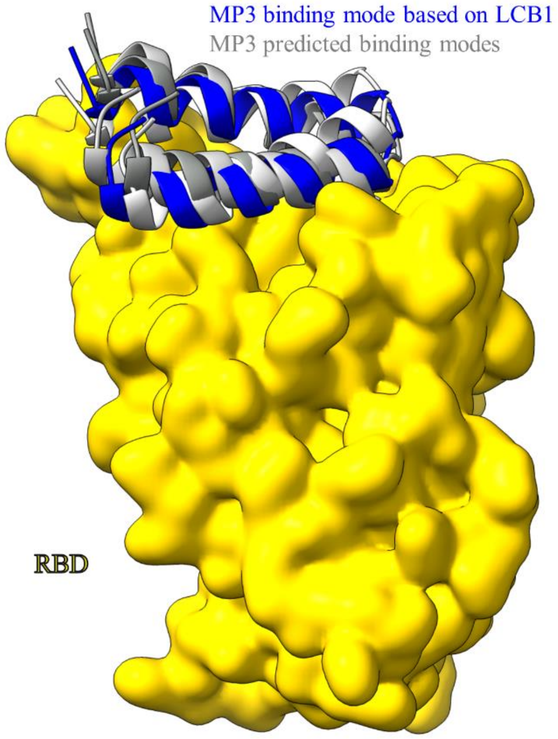

Figure S4. Binding mode of best predicted MP3:RBD complexes using ZDOCK server [22] (gray color) vs LCB1:RBD complex from Cao et al. study (blue color). Note we show only the H1 and H2 of LCB1.

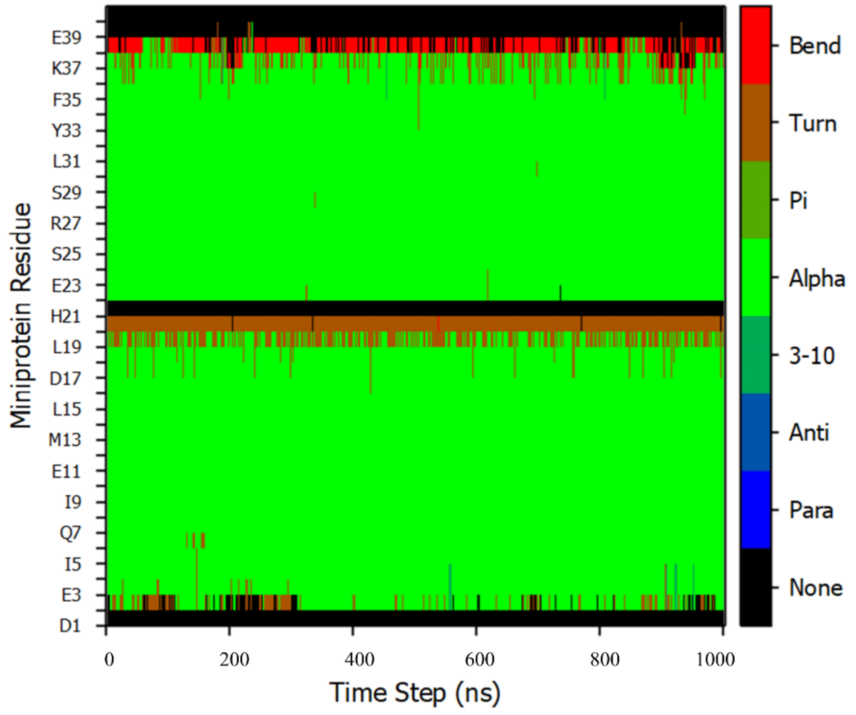

Figure S5. The secondary structure content of MP3 through 1 μs MD simulation.

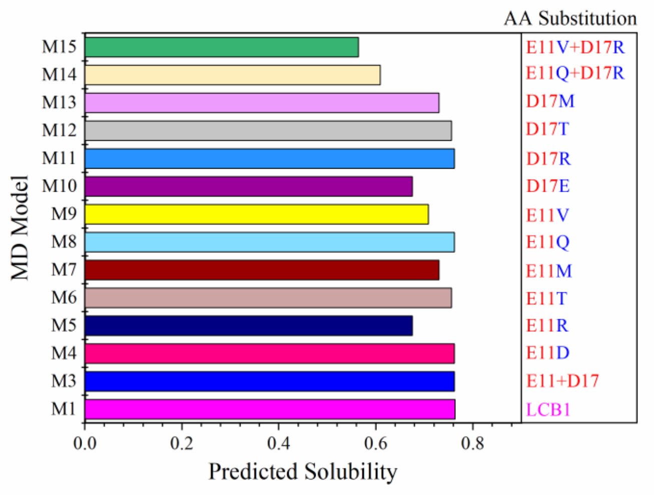

Figure S6. Predicted solubility of miniproteins using Protein-sol web server [23]. According to this web server, the population average for the experimental dataset is 0.45, hence any scaled solubility value more than 0.45 is expected to have a higher solubility. Each MP has a solubility greater than 0.45.

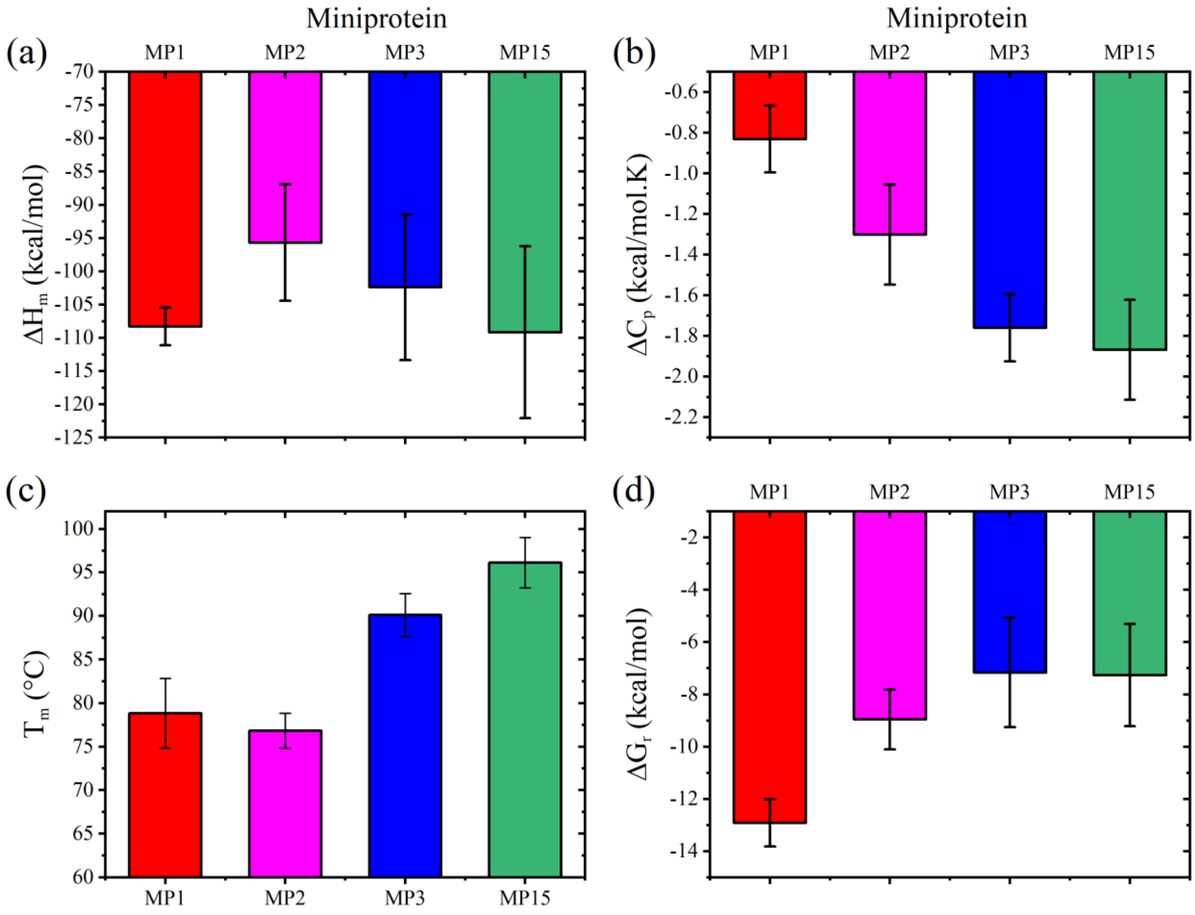

Figure S7. Prediction values of the thermodynamic quantities associated with the folding transition of miniproteins using the SCooP algorithm [24]. (**a**) Standard folding enthalpy (ΔH_m) measured at melting temperature (T_m); (**b**) the standard folding heat capacity (ΔC_p); (**c**) melting temperature (T_m); and (**d**) Reference folding free energy at room temperature (ΔG_r).

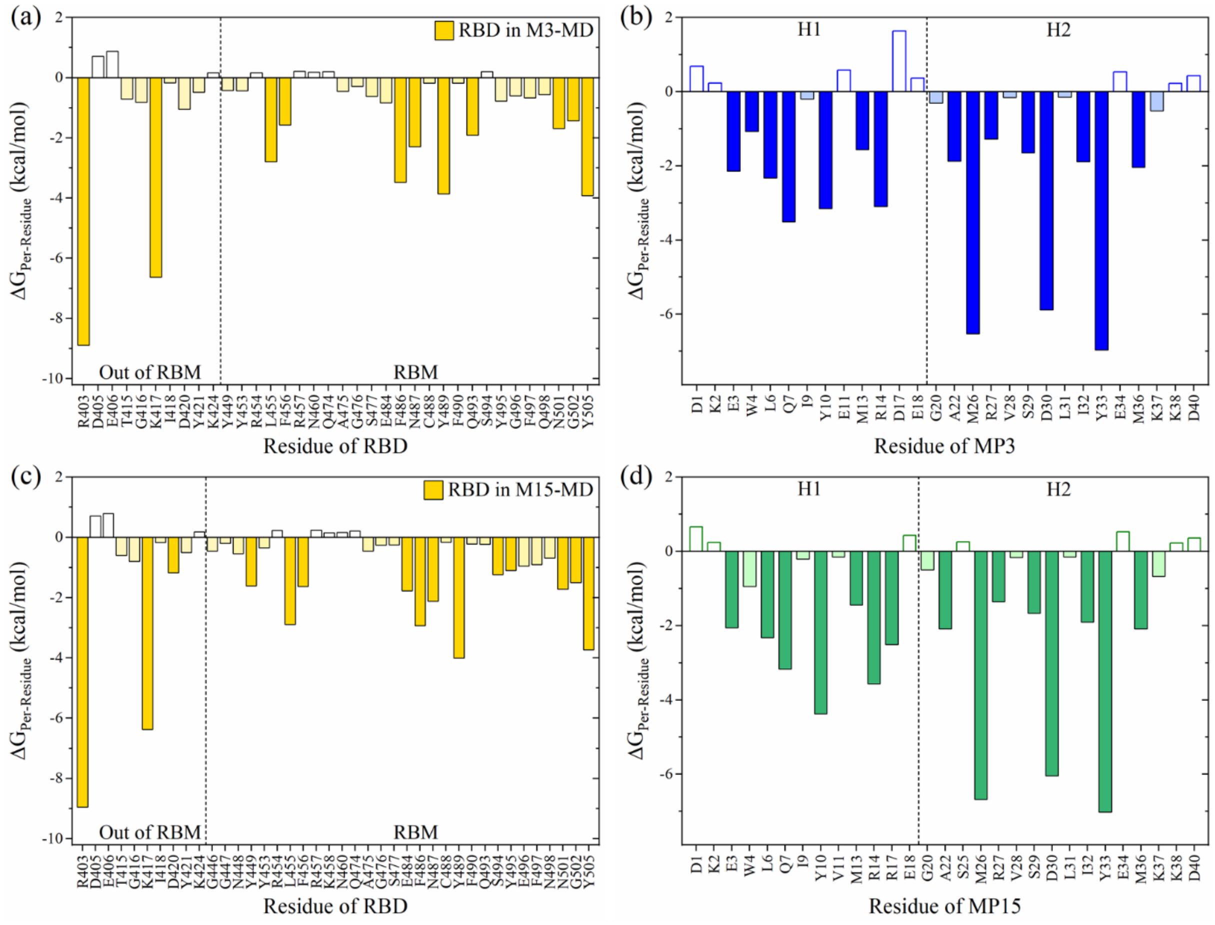

Figure S8. Per-residue interaction spectrum of M3-MD (**a**,**b**) and M15-MD (**c**,**d**) models. The left panels are for RBD residues while the right panels are for miniprotein residues.

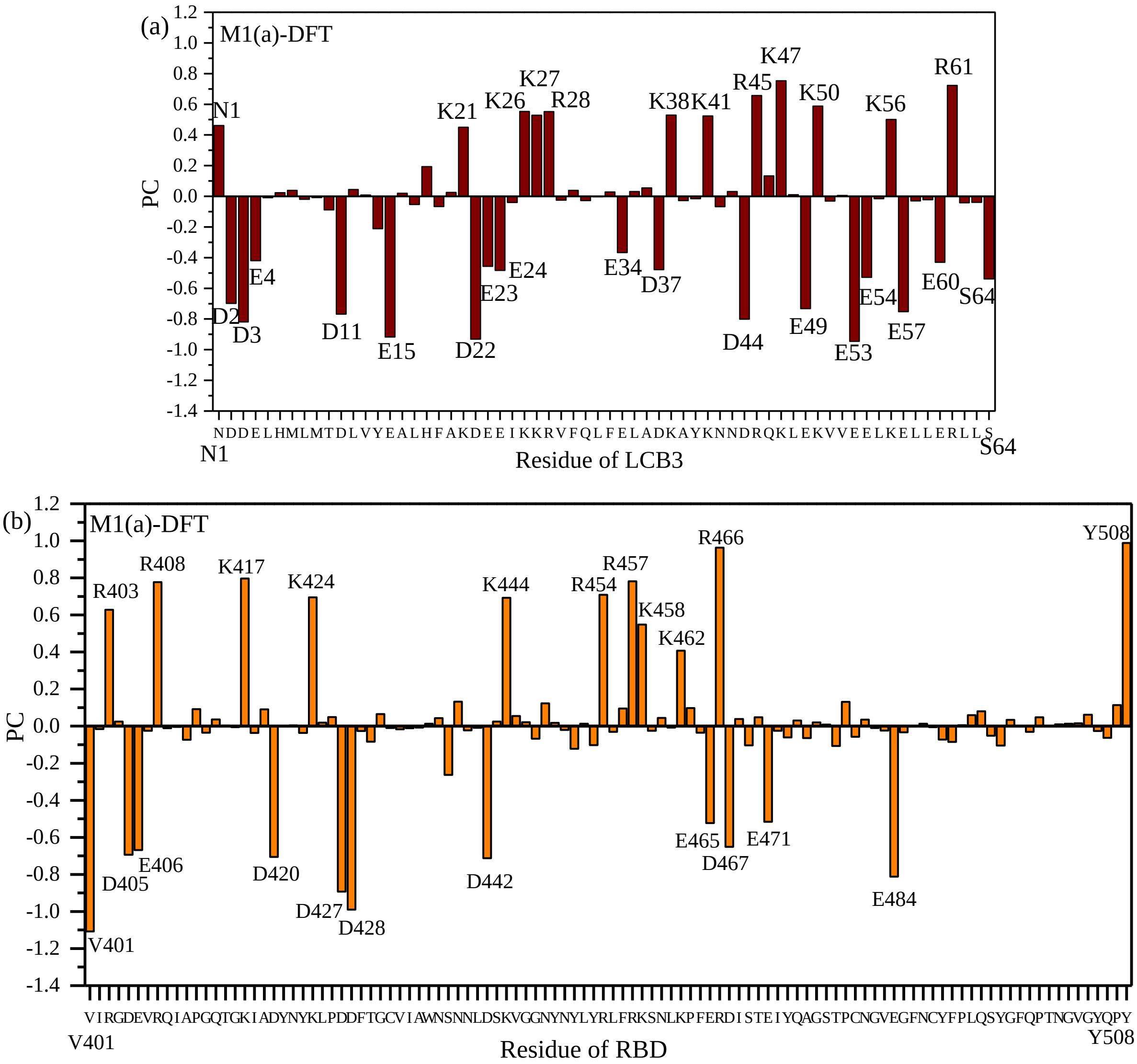

Figure S9. Bar graph with PC distribution for (**a**) LCB3 and (**b**) RBD of M1(a)-DFT model. AAs with reasonable large positive and negative PC are marked.

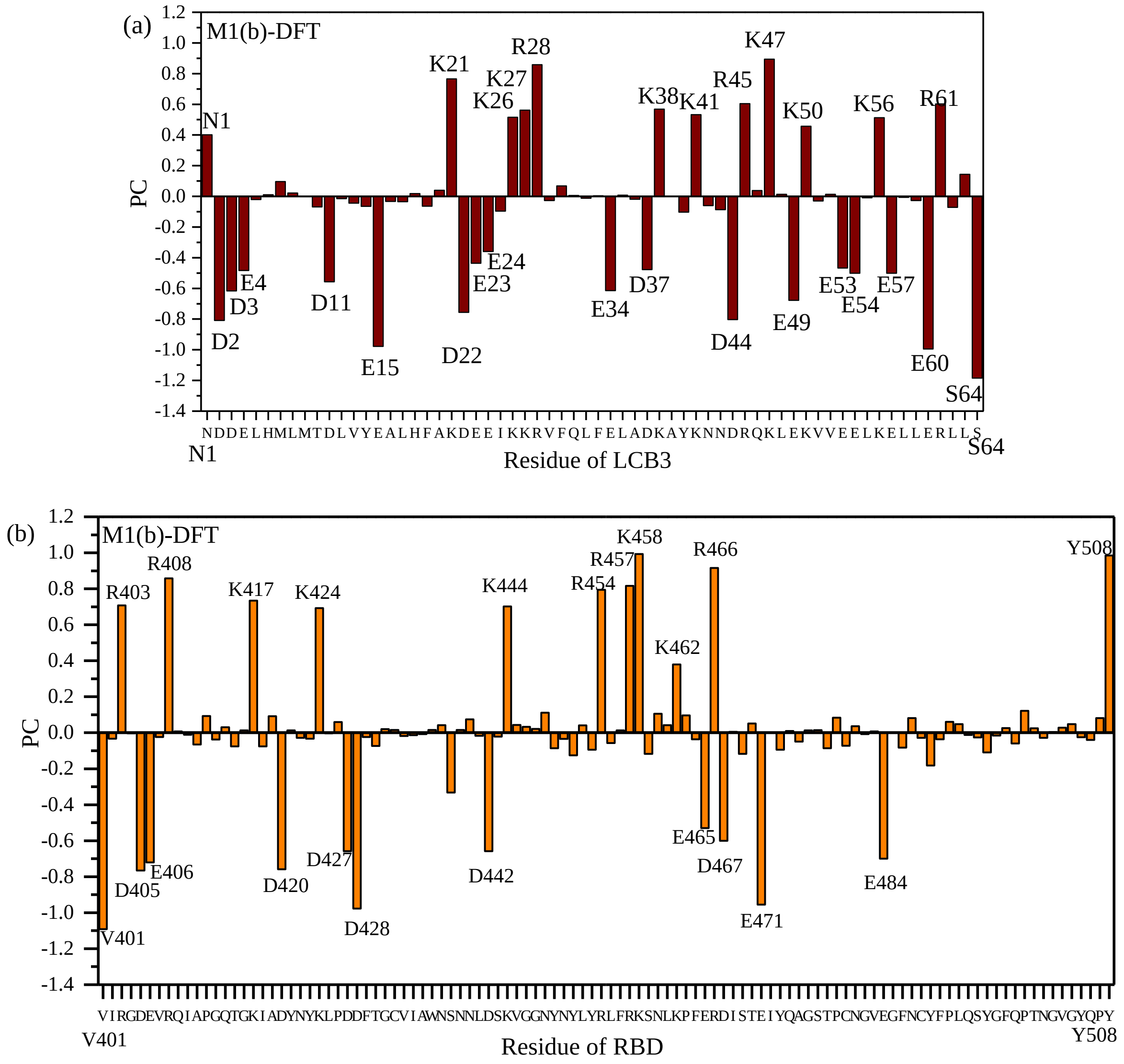

Figure S10. Bar graph with PC distribution for (**a**) LCB3 and (**b**) RBD of M1(b)-DFT model. AAs with reasonable large positive and negative PC are marked.

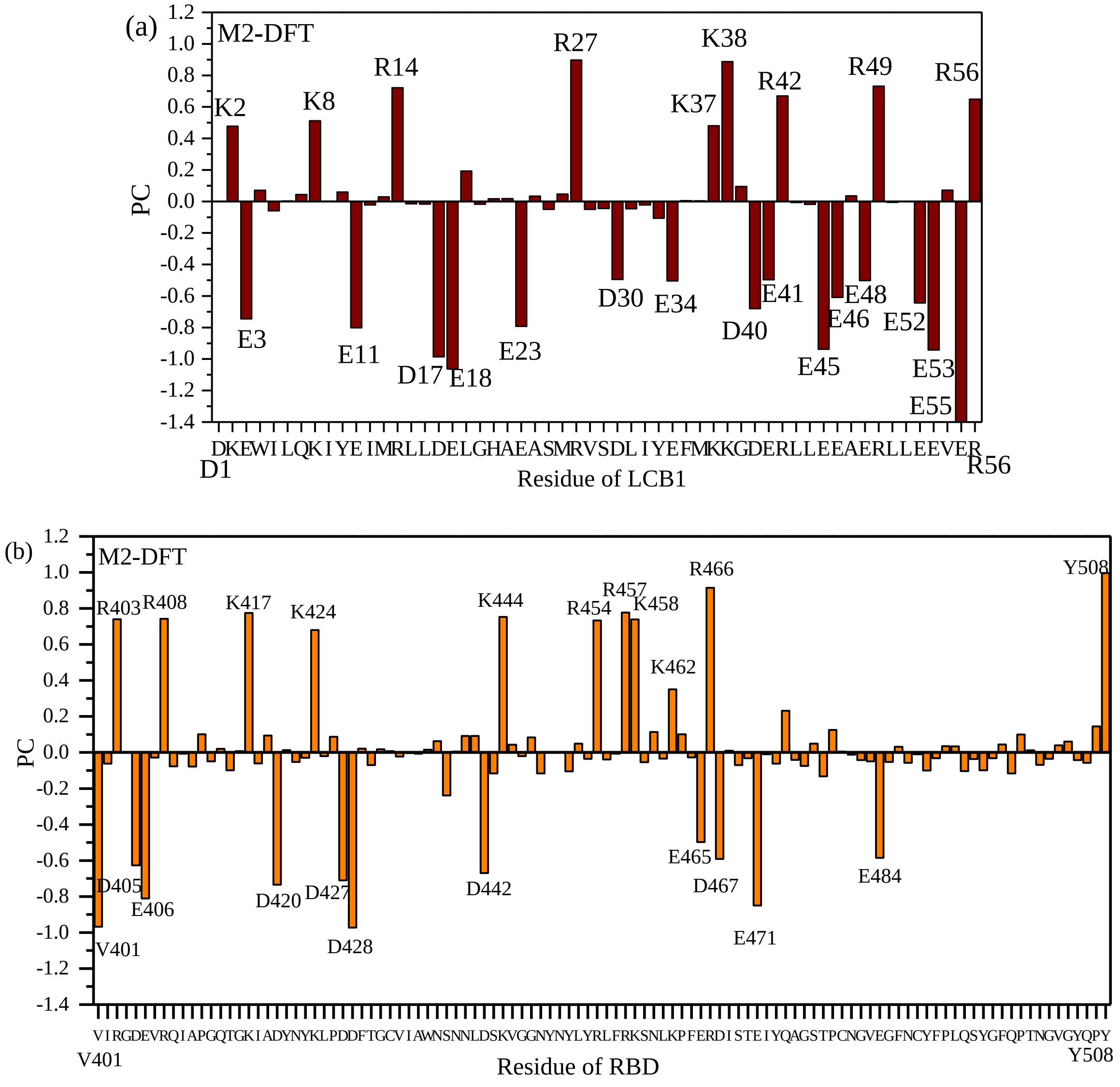

Figure S11. Bar graph with PC distribution for (**a**) LCB1 and (**b**) RBD of M2-DFT model. AAs with reasonable large positive and negative PC are marked.

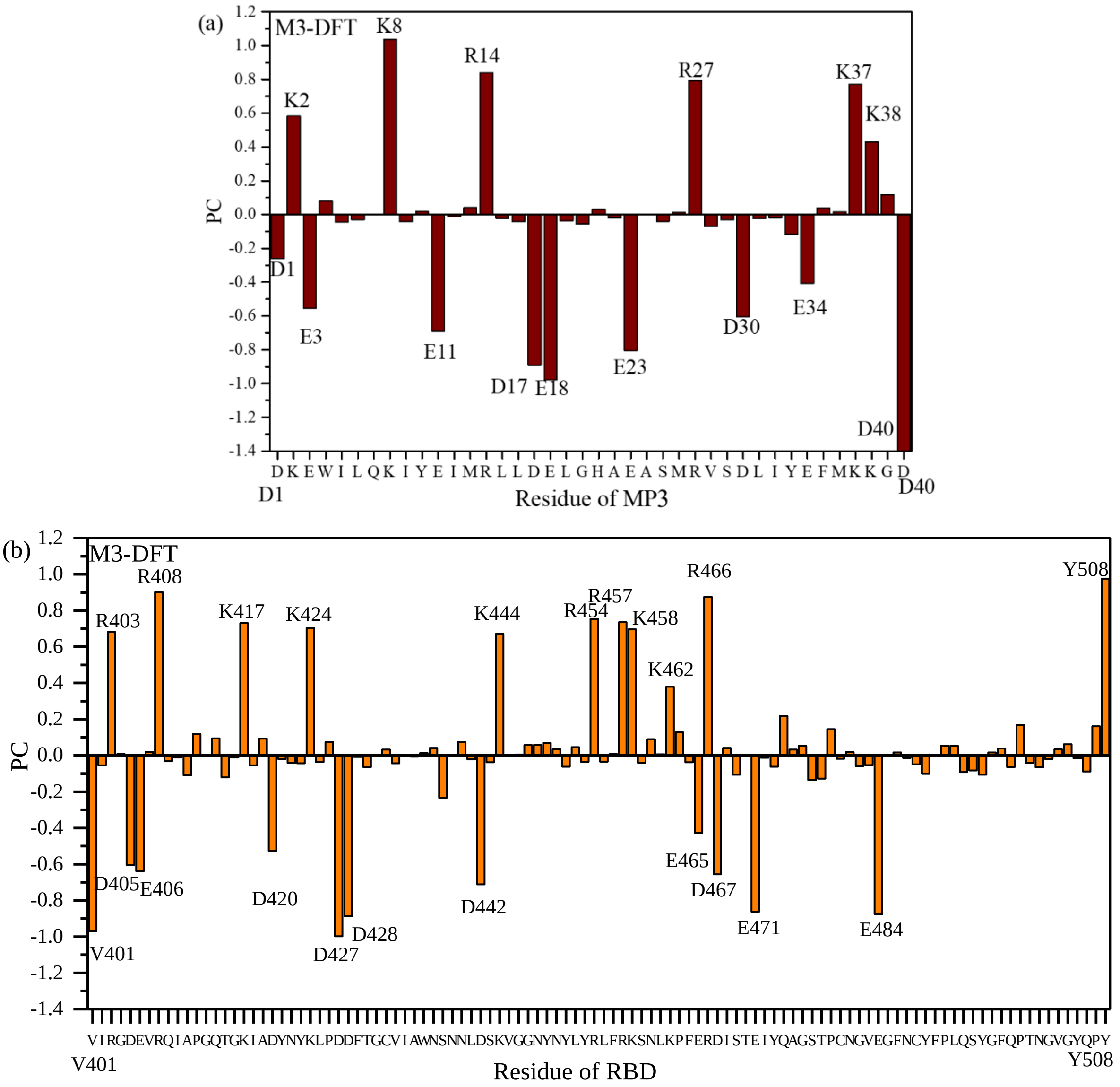

Figure S12. Bar graph with PC distribution for (**a**) MP3 and (**b**) RBD of M3-DFT model. AAs with reasonable large positive and negative PC are marked.

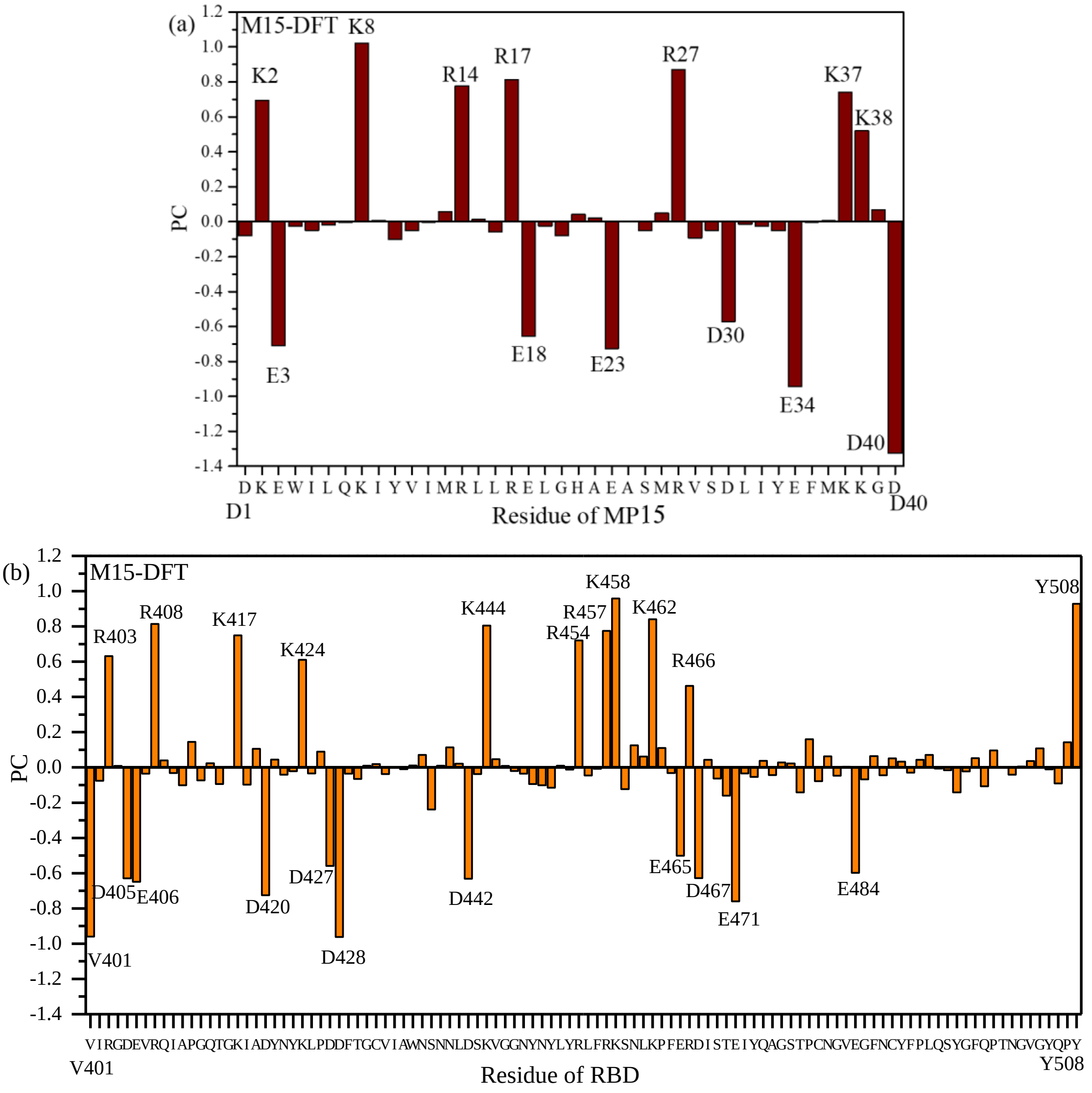

Figure S13. Bar graph with PC distribution for (**a**) MP15 and (**b**) RBD of M15-DFT model. AAs with reasonable large positive and negative PC are marked.

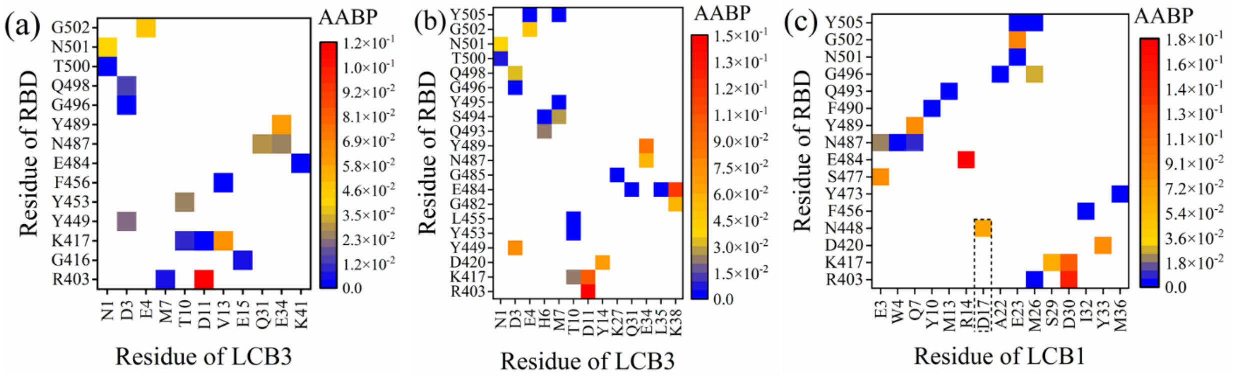

Figure S14. AA-AA bond pair (AABP) for (**a**) M1(a)-DFT, (**b**) M1(b)-DFT, and M2-DFT (**c**) models. Each square cell represents the intersection AA from RBD on the vertical axis and AA from mini-protein on the horizontal axis. These pairs have different strengths based on AABP values, the strongest pair is the one with highest AABP and vice versa. By comparing (a) and (b), RBD conformational changes induce more interactions with LCB3. The overall AABP of (**a**–**c**) are 0.4664 e, 0.9242 e and 0.9881 e respectively, indicating that LCB1 binds RBD stronger than LCB3.

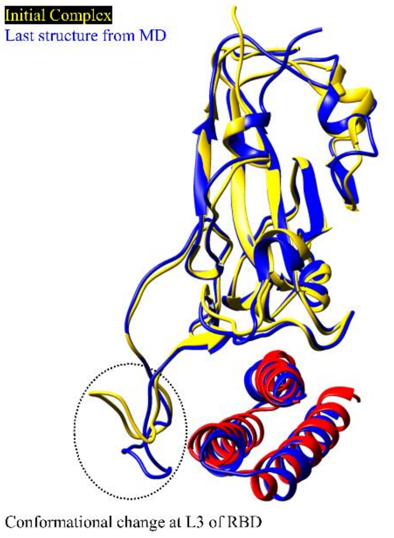

Figure S15. Alignment between the M1-MD model before MD simulation (RBD in yellow and LCB3 in red) and the last step from MD simulation (both proteins in blue). There is a conformational change at the Loop 3 (L3) of RBD between two conformations.

References

1. Miyamoto, S.; Kollman, P.A. Settle: An analytical version of the SHAKE and RATTLE algorithm for rigid water models. *J. Comput. Chem.* **1992**, *13*, 952–962.
2. Darden, T.; York, D.; Pedersen, L. Particle mesh Ewald: An N · log (N) method for Ewald sums in large systems. *J. Chem. Phys.* **1993**, *98*, 10089–10092.
3. Gotz, A.W.; Williamson, M.J.; Xu, D.; Poole, D.; Le Grand, S.; Walker, R.C. Routine microsecond molecular dynamics simulations with AMBER on GPUs. 1. Generalized born. *J. Chem. Theory Comput.* **2012**, *8*, 1542–1555.
4. Salomon-Ferrer, R.; Go¨tz, A.W.; Poole, D.; Le Grand, S.; Walker, R.C. Routine microsecond molecular dynamics simulations with AMBER on GPUs. 2. Explicit solvent particle mesh Ewald. *J. Chem. Theory Comput.* **2013**, *9*, 3878–3888.
5. Miller, B.R., III; McGee Jr, T.D.; Swails, J.M.; Homeyer, N.; Gohlke, H.; Roitberg, A.E. MMPBSA. py: An efficient program for end-state free energy calculations. *J. Chem. Theory Comput.* **2012**, *8*, 3314–3321.
6. Wang, E.; Sun, H.; Wang, J.; Wang, Z.; Liu, H.; Zhang, J.Z.; Hou, T. End-point binding free energy calculation with MM/PBSA and MM/GBSA: Strategies and applications in drug design. *Chem. Rev.* **2019**, *119*, 9478–9508.
7. Jawad, B.; Poudel, L.; Podgornik, R.; Steinmetz, N.F.; Ching, W.Y. Molecular mechanism and binding free energy of doxorubicin intercalation in DNA. *Phys. Chem. Chem. Phys.* **2019**, *21*, 3877–3893.
8. Jawad, B.; Poudel, L.; Podgornik, R.; Ching, W.Y. Thermodynamic Dissection of the Intercalation Binding Process of Doxorubicin to dsDNA with Implications of Ionic and Solvent Effects. *J. Phys. Chem. B* **2020**, *124*, 7803–7818.
9. Onufriev, A.; Bashford, D.; Case, D.A. Modification of the generalized born model suitable for macromolecules. *J. Phys. Chem. B* **2000**, *104*, 3712–3720.
10. VASP—Vienna Ab initio Simulation Package, https://www.vasp.at/.
11. Perdew, J.P.; Burke, K.; Ernzerhof, M. Generalized gradient approximation made simple. *Phys. Rev. Lett.* **1996**, *77*, 3865.
12. Ching, W.-Y.; Rulis, P. Electronic Structure Methods for Complex Materials: The Orthogonalized Linear Combination of Atomic Orbitals. Oxford University Press: Oxford, UK, 2012.
13. Adhikari, P.; Podgornik, R.; Jawad, B.; Ching, W.Y. First-Principles Simulation of Dielectric Function in Biomolecules. *Materials* **2021**, *14*, 5774.
14. Baral, K.; Adhikari, P.; Jawad, B.; Podgornik, R.; Ching, W.Y. Solvent Effect on the Structure and Properties of RGD Peptide (1FUV) at Body Temperature (310 K) Using Ab Initio Molecular Dynamics. *Polymers* **2021**, *13*, 3434.
15. Poudel, L.; Twarock, R.; Steinmetz, N.F.; Podgornik, R.; Ching, W.-Y. Impact of Hydrogen Bonding in the Binding Site between Capsid Protein and Ms2 Bacteriophage Ssrna. *J. Phys. Chem. B* **2017**, *121*, 6321–6330.
16. Adhikari, P.; Li, N.; Shin, M.; Steinmetz, N.F.; Twarock, R.; Podgornik, R.; Ching, W.Y. Intra-and intermolecular atomic-scale interactions in the receptor binding domain of SARS-CoV-2 spike protein: Implication for ACE2 receptor binding. *Phys. Chem. Chem. Phys.* **2020**, *22*, 18272–18283.
17. Ching, W.Y.; Adhikari, P.; Jawad, B.; Podgornik, R. Ultra-large-scale ab initio quantum chemical computation of bio-molecular systems: The case of spike protein of SARS-CoV-2 virus. *Comput. Struct. Biotechnol. J.* **2021**, *19*, 1288–1301.
18. Adhikari, P.; Ching, W.Y. Amino acid interacting network in the receptor-binding domain of SARS-CoV-2 spike protein. *RSC Adv.* **2020**, *10*, 39831–39841.
19. Jawad, B.; Adhikari, P.; Podgornik, R.; Ching, W.-Y. Key Interacting Residues between RBD of SARS-CoV-2 and ACE2 Receptor: Combination of Molecular Dynamic Simulation and Density Functional Calculation. *J. Chem. Inf. Modeling* **2021**, *61*, 4425–4441.

20. Mulliken, R.S. Electronic Population Analysis on Lcao–Mo Molecular Wave Functions. I. *J. Chem. Phys.* **1955**, *23*, 1833–1840.
21. Mulliken, R. Electronic Population Analysis on Lcao–Mo Molecular Wave Functions. Ii. Overlap Populations, Bond Orders, and Covalent Bond Energies. *J. Chem. Phys.* **1955**, *23*, 1841–1846.
22. Pierce, B.G.; Wiehe, K.; Hwang, H.; Kim, B.H.; Vreven, T.; Weng, Z. ZDOCK server: Interactive docking prediction of protein–protein complexes and symmetric multimers. *Bioinformatics* **2014**, *30*, 1771–1773.
23. Hebditch, M.; Carballo-Amador, M.A.; Charonis, S.; Curtis, R.; Warwicker, J. Protein–Sol: A web tool for predicting protein solubility from sequence. *Bioinformatics* **2017**, *33*, 3098–3100.
24. Pucci, F.; Kwasigroch, J.M.; Rooman, M. SCooP: An accurate and fast predictor of protein stability curves as a function of temperature. *Bioinformatics* **2017**, *33*, 3415–3422.
